# Transgenerational inheritance of diabetes susceptibility in male offspring with maternal androgen exposure

**DOI:** 10.1038/s41421-025-00769-1

**Published:** 2025-02-12

**Authors:** Yuqing Zhang, Shourui Hu, Shan Han, Congcong Liu, Xiaofan Liang, Yuxuan Li, Zongxuan Lin, Yiming Qin, Chunxuan Geng, Yue Liu, Linlin Cui, Jingmei Hu, Changming Zhang, Zhao Wang, Xin Liu, Jinlong Ma, Zi-Jiang Chen, Han Zhao

**Affiliations:** 1 State Key Laboratory of Reproductive Medicine and Offspring Health, Center for Reproductive Medicine, Institute of Women, Children and Reproductive Health, the Second Hospital, Shandong University, Jinan, Shandong China; 2 National Research Center for Assisted Reproductive Technology and Reproductive Genetics, Shandong University, Jinan, Shandong China; 3 Key Laboratory of Reproductive Endocrinology of Ministry of Education (Shandong University), Ministry of Education, Jinan, Shandong China; 4 Shandong Technology Innovation Center for Reproductive Health, Jinan, Shandong China; 5 Shandong Provincial Clinical Research Center for Reproductive Health, Jinan, Shandong China; 6 Shandong Key Laboratory of Reproductive Research and Birth Defect Prevention, Jinan, Shandong China; 7 Research Unit of Gametogenesis and Health of ART-Offspring, Chinese Academy of Medical Sciences (No.2021RU001), Jinan, Shandong China; 8 Shanghai Key Laboratory for Assisted Reproduction and Reproductive Genetics, Shanghai, China; 9 Department of Reproductive Medicine, Ren Ji Hospital, Shanghai Jiao Tong University School of Medicine, Shanghai, China

**Keywords:** Mechanisms of disease, DNA methylation

## Abstract

Androgen exposure (AE) poses a profound health threat to women, yet its transgenerational impacts on male descendants remain unclear. Here, employing a large-scale mother-child cohort, we show that maternal hyperandrogenism predisposes sons to β-cell dysfunction. Male offspring mice with prenatal AE exhibited hyperglycemia and glucose intolerance across three generations, which were further exacerbated by aging and a high-fat diet. Mechanistically, compromised insulin secretion underlies this transgenerational susceptibility to diabetes. Integrated analyses of methylome and transcriptome revealed differential DNA methylation of β-cell functional genes in AE-F1 sperm, which was transmitted to AE-F2 islets and further retained in AE-F2 sperm, leading to reduced expression of genes related to insulin secretion, including *Pdx1*, *Irs1*, *Ptprn2*, and *Cacna1c*. The methylation signatures in AE-F1 sperm were corroborated in diabetic humans and the blood of sons with maternal hyperandrogenism. Moreover, caloric restriction and metformin treatments normalized hyperglycemia in AE-F1 males and blocked their inheritance to offspring by restoring the aberrant sperm DNA methylations. Our findings highlight the transgenerational inheritance of impaired glucose homeostasis in male offspring from maternal AE via DNA methylation changes, providing methylation biomarkers and therapeutic strategies to safeguard future generations’ metabolic health.

## Introduction

The global burden of metabolic diseases, particularly among children and adolescents, has surged in recent decades, with the highest mortality attributed to type 2 diabetes (T2D) and obesity^1^. Growing evidence suggests that the risk of developing these conditions is primed early in life by exposure to adverse intrauterine environments^2,3^. This phenomenon, known as the Developmental Origins of Health and Disease (DOHaD), contributes to the metabolic programming and subsequent susceptibility of poor metabolic health later in adulthood — a risk that can be inherited across multiple generations^4^. These suboptimal prenatal exposures that range from hormonal and nutritional imbalances to toxicants and endocrine disruptors, are linked to varied susceptibilities to metabolic disorders among offspring^5^. These emerging data highlight the enduring and profound effects of prenatal exposures on the long-term metabolic health of their offspring, raising the possibility that the current epidemic of metabolic diseases might be rooted in developmental programming.

Perturbations in the maternal hormone milieu, especially sex steroids, are recognized as driving factors to adverse developmental programming^6^. Among these hormones, androgens are implicated in the etiology of T2D and are associated with metabolic dysfunctions in women^7^. Maternal androgen levels are shaped by the combined effects of exogenous environmental exposures and endogenous diseases. Exogenous androgen excess includes overuse of androgenic drugs and exposure to steroid mimetic chemicals such as bisphenol A and perfluorooctanoic acid that are ubiquitously present in the environment^8,9^. Endogenous hyperandrogenism can stem from prevalent endocrine diseases like polycystic ovary syndrome (PCOS), congenital adrenal hyperplasia, Cushing’s syndrome, or androgen-secreting tumors^10^, affecting 20%‒28% of women globally^11,12^. Moreover, epidemiological studies demonstrated racial and ethnic disparities in gestational androgen levels, with Hispanic, African-American, and South Asian women exhibiting higher levels than others^13,14^, correlating with increased diabetes prevalence^15,16^. To be noted, women affected by androgen excess frequently experience a series of metabolic derangements, including obesity, insulin resistance, and pancreatic β-cell dysfunction, substantially increasing the risk of developing impaired glucose tolerance and T2D^17^. Their daughters are also more prone to developing reproductive disorders in adulthood^18^. Animal studies indicate that maternal androgen excess instigates transgenerational PCOS-like traits in female offspring without glucose disturbance. Despite these insights, the transgenerational metabolic consequences of maternal hyperandrogenism, particularly in male descendants, remain poorly understood.

In the present study, leveraging a clinical mother-child cohort and a transgenerational mouse model, we systematically assessed the inter- and transgenerational effect of maternal androgen exposure (AE) on the metabolism of male offspring. We found that altered DNA methylation that affects insulin secretion underlies the transgenerational inheritance of hyperglycemia. Moreover, we report that caloric restriction (CR) and metformin (Met) treatments can prevent the transgenerational inheritance of glucose disturbance, providing promising interventions for enhancing the metabolic well-being of future generations.

## Results

### Maternal hyperandrogenism induces inter- and trans-generational glucose metabolic disturbances in male offspring at an early age

**Fig. 1:**
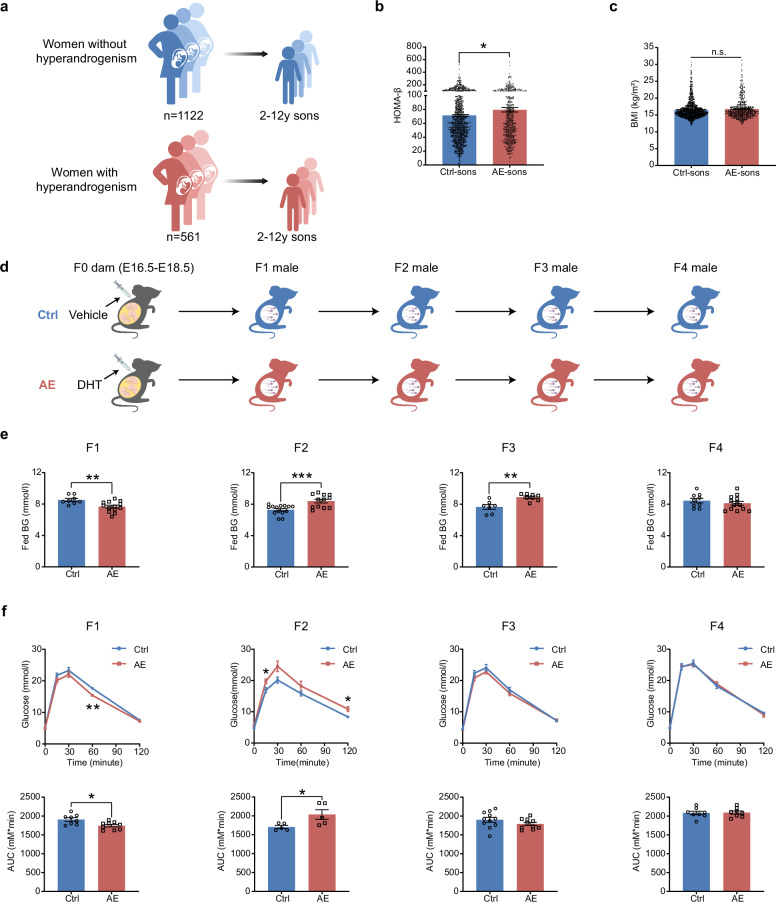
Maternal hyperandrogenism leads to inter- and transgenerational glucose metabolic disturbances in male offspring at early-life stages. **a** The cohort of sons born to women with hyperandrogenism (*n* = 561) and without hyperandrogenism (*n* = 1122) with the ages of 2 to 12 years old. **b, c** The levels of HOMA-β (**b**) and BMI (**c**) in sons born to control mothers and sons born to hyperandrogenic mothers. **d** Schematic illustration of mouse experimental design and offspring breeding from control (Ctrl) and AE groups. **e** Fed blood glucose levels in F1‒F4 male offspring at 8 weeks of age. Ctrl (F1: *n* = 8; F2: *n* = 14; F3: *n* = 7; F4: *n* = 9); AE (F1: *n* = 13; F2: *n* = 12; F3: *n* = 8; F4: *n* = 13). **f** IPGTT of F1‒F4 male offspring at 8 weeks of age and the corresponding AUC of glucose levels. Ctrl (F1: *n* = 8; F2: *n* = 5; F3: *n* = 11; F4: *n* = 8); AE (F1: *n* = 9; F2: *n* = 5; F3: *n* = 10; F4: *n* = 8). Data are presented as mean ± SEM. *P < 0.05, **P < 0.01, ***P < 0.001, ns, non-significant. Significance is assessed by two-tailed unpaired Student’s *t*-test.

Based on our birth cohort at the Center for Reproductive Medicine, Shandong University, we assessed whether sons born to women with hyperandrogenism were more susceptible to developing metabolic abnormalities. A total of 561 sons born to mothers with hyperandrogenism and 1122 sons born to mothers without hyperandrogenism were enrolled (Fig. 1a). Analysis of metabolic parameters revealed that sons from hyperandrogenism mothers exhibited significantly elevated levels of homeostasis model for β-cell function (HOMA-β) compared to their counterparts (mean 79.612 vs 71.248, *P* < 0.05) (Fig. 1b), with comparable body mass index (BMI) at this childhood stage (Fig. 1c). Maternal clinical characteristics are detailed in Supplementary Table S1. The follow-up ages of these sons ranged from 2 to 12 years, with an average age of 5 years for both groups (Supplementary Table S2). These results strongly suggest an elevated risk of pancreatic β-cell dysfunction in sons born to women with hyperandrogenism, highlighting the intergenerational impact of maternal hormonal status on offspring metabolic health.

Then we employed mouse model to test whether male offspring mice from maternal androgen-exposed dams could replicate the metabolic disturbances observed in sons of maternal hyperandrogenism, and whether these effects could be transmitted transgenerationally. As in utero exposure exerts direct effects on embryos, only phenotypes manifest in subsequent generations (beyond F1) of male lineage are considered as transgenerational inheritance^19,20^. Pregnant dams (F0) were injected subcutaneously with dihydrotestosterone from embryonic day E16.5 to E18.5, establishing a prenatal AE mouse model^18,21^. F0 dams received vehicle alone at the same time were used as controls. The born male offspring (F1) were maintained unexposed and then bred with wild-type female mice to produce second-generation (F2) progeny. This breeding strategy was further conducted to yield third (F3) and fourth (F4) generation offspring (Fig. 1d), then metabolic phenotypes were monitored up to the F4 generation.

At 8 weeks of age, F1 male offspring from the AE group exhibited significantly lower fed blood glucose levels compared to controls (Fig. 1e), suggesting enhanced β-cell function at this early life stage. This initial adaptation, however, shifts in AE-F2 males, where a significant increase in fed blood glucose levels emerged, a trait further transmitted to AE-F3 offspring (Fig. 1e), indicating a transgenerational hyperglycemia phenotype. Despite these fluctuations, body weight, food intake, and fasting blood glucose levels remained consistent across both groups and generations (Supplementary Fig. S1a‒c). Intriguingly, at the same age of 8 weeks, AE-F1 males also displayed significantly reduced blood glucose levels and decreased area under the curve (AUC) during the intraperitoneal glucose tolerance test (IPGTT) (Fig. 1f), indicating a temporary resilience against glucose intolerance. However, AE-F2 males showed significantly increased blood glucose levels as well as augmented AUC during IPGTT at 8 weeks of age, marking a prediabetes status; this difference was not observed in young F3 and F4 males (Fig. 1f). These findings collectively suggest that prenatal AE may impair the inter- and transgenerational glucose metabolic homeostasis in male progeny at a young age.

To determine the whole-body energy utilization, we used indirect calorimetry with metabolic cages and found that F1, but not F2, male mice from the AE group showed significantly decreased respiratory exchange ratio (Supplementary Fig. S1d), without any changes in energy expenditure (Supplementary Fig. S1e). These data indicated reduced carbohydrate utilization exclusively in AE-F1 males that does not extend to subsequent generations. Further examinations showed no significant differences in fat mass, adipose tissue morphology or adipocyte size across F1 to F3 male offspring (Supplementary Fig. S1f‒h).

### Advanced age and obesity act as second strikes to exacerbate the hyperglycemia and glucose intolerance in F1‒F3 male mice with prenatal AE

**Fig. 2:**
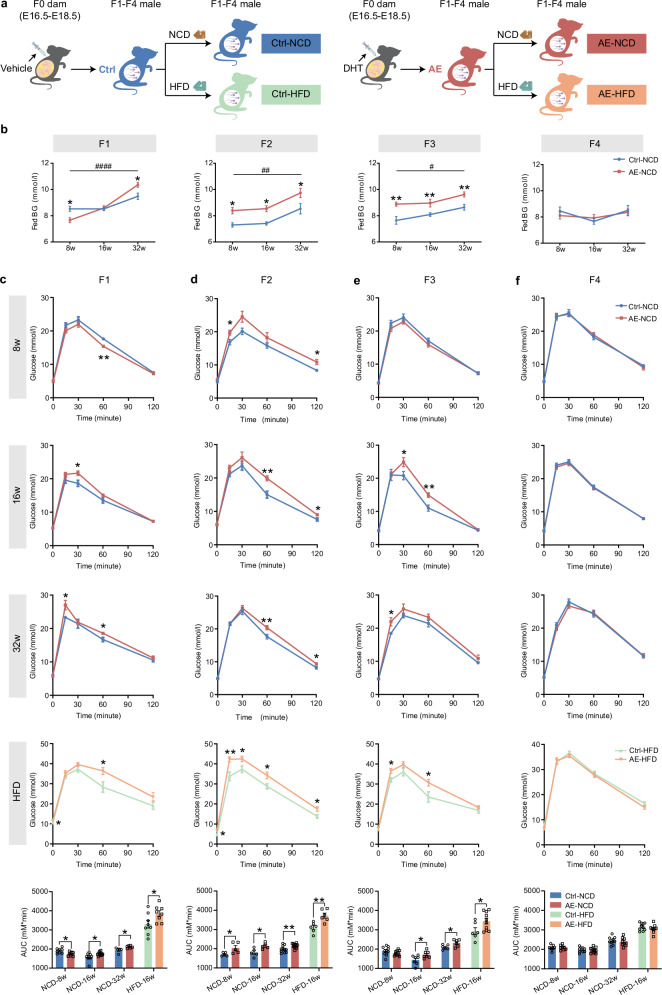
Prenatal AE causes transgenerational hyperglycemia and glucose intolerance in F1‒F3 male offspring that are aggravated by advanced age and high-fat diet. **a** Schematic illustration of the experimental design of the mouse models. **b** Fed blood glucose levels in F1‒F4 male offspring at the indicated ages. Ctrl-NCD-8w (F1: *n* = 8; F2: *n* = 14; F3: *n* = 7; F4: *n* = 9); AE-NCD-8w (F1: *n* = 13; F2: *n* = 12; F3: *n* = 8; F4: *n* = 13); Ctrl-NCD-16w (F1: *n* = 11; F2: *n* = 13; F3: *n* = 15; F4: *n* = 10); AE-NCD-16w (F1: *n* = 15; F2: *n* = 12; F3: *n* = 9; F4: *n* = 13); Ctrl-NCD-32w (F1: *n* = 11; F2: *n* = 11; F3: *n* = 15; F4: *n* = 9); AE-NCD-32w (F1: *n* = 10; F2: *n* = 14; F3: *n* = 10; F4: *n* = 11). **c** IPGTT of F1 male offspring at the indicated ages and the corresponding AUC of glucose levels. Ctrl (NCD-8w: *n* = 8; NCD-16w: *n* = 9; NCD-32w: *n* = 5; HFD-16w: *n* = 8); AE (NCD-8w: *n* = 9; NCD-16w: *n* = 11; NCD-32w: *n* = 6; HFD-16w: *n* = 8). **d** IPGTT of F2 male offspring at the indicated ages and the corresponding AUC of glucose levels. Ctrl (NCD-8w: *n* = 5; NCD-16w: *n* = 5; NCD-32w: *n* = 11; HFD-16w: *n* = 6); AE (NCD-8w: *n* = 5; NCD-16w: *n* = 5; NCD-32w: *n* = 12; HFD-16w: *n* = 6). **e** IPGTT of F3 male offspring at the indicated ages and the corresponding AUC of glucose levels. Ctrl (NCD-8w: *n* = 11; NCD-16w: *n* = 7; NCD-32w: *n* = 6; HFD-16w: *n* = 6); AE (NCD-8w: *n* = 10; NCD-16w: *n* = 7; NCD-32w: *n* = 6; HFD-16w: *n* = 10). **f** IPGTT of F4 male offspring at the indicated ages and the corresponding AUC of glucose levels. Ctrl (NCD-8w: *n* = 8; NCD-16w: *n* = 9; NCD-32w: *n* = 10; HFD-16w: *n* = 10); AE (NCD-8w: *n* = 8; NCD-16w: *n* = 12; NCD-32w: *n* = 9; HFD-16w: *n* = 9). Data are presented as mean ± SEM. *, Ctrl vs AE; statistical analyses of **b, c, d, e**, and **f** are performed using two-tailed unpaired Student’s *t*-test. #, AE mice at different ages; statistical analyses of **b** # are performed using one-way ANOVA. *P < 0.05, #*P* < 0.05, **P < 0.01, ##*P* < 0.01, ####*P* < 0.0001.

Given the early onset of metabolic disturbances in male offspring from maternal AE, we next explored whether these phenotypes were susceptible to the metabolic challenge increasing with age. For this purpose, we monitored glucose metabolism in F1‒F4 male mice across different life stages under normal chow diet (NCD), from 8 to 32 weeks of age (Fig. 2a upper panel; Supplementary Fig. S2a). Surprisingly, the fed blood glucose levels in AE-F1 males escalated with age, initially presenting lower levels at 8 weeks, aligning with those of controls at 16 weeks, then turning to significantly higher levels by 32 weeks of age (mean 10.4 mM vs 9.5 mM of control) (Fig. 2b). This trend of progressively increasing fed blood glucose with advanced age in AE males persists in F2 and F3 generations (Fig. 2b), suggesting a transgenerational effect.

Moreover, in contrast to improved glucose tolerance at 8 weeks, AE-F1 males began to show significantly higher blood glucose levels during IPGTT, along with an increased AUC starting at 16 weeks of age, with this deterioration becoming even more pronounced by 32 weeks (Fig. 2c). In the F2 generation, adult AE males exhibited significantly impaired glucose tolerance at all assessed ages (Fig. 2d). AE-F2 female mice also showed significant glucose intolerance, consistent with F2 males (Supplemetary Fig. S2b). For the F3 generation, although glucose tolerance appeared normal at 8 weeks, AE males began to display significantly elevated blood glucose levels during IPGTT from 16 weeks onwards, with these differences becoming more evident by 32 weeks (Fig. 2e). This pattern of glucose intolerance was not observed in AE-F4 males (Fig. 2f). These longitudinal findings suggest a progressive exacerbation of the hyperglycemic phenotype in AE males with advanced age.

Obesity represents another critical environmental stress for metabolic homeostasis^22^. To evaluate the combined impact of diet-induced obesity with prenatal AE, offspring mice in each generation were subjected to a high-fat diet (HFD), with mating and subsequent breeding only restricted to NCD-fed lineages (Fig. 2a). Following HFD administration, AE-F1 mice exhibited a significant increase in fed hyperglycemia compared to controls, reaching levels up to 11.6 mM compared to 10.6 mM in controls, even though body weights were similar in both groups (Supplementary Fig. S2c, d). This worsened hyperglycemic state was also observed in AE-F2 males (Supplementary Fig. S2d, e). Moreover, F1 males in the AE group showed significantly compromised glucose tolerance compared to non-AE controls upon HFD feeding (Fig. 2c). This impaired glucose tolerance was not only more severe in the context of HFD but also transmitted to the F3 generation (Fig. 2d, e). The differences in blood glucose levels and glucose intolerance were no longer observable in F4 males on HFD (Fig. 2f; Supplementary Fig. S2d), suggesting that the germline of F3 mice might have lost the inheritance information of hyperglycemic traits. Taken together, these data demonstrate that male offspring from maternal AE are predisposed to developing transgenerational diabetes, which is exacerbated by further strikes of aging and HFD, highlighting the synergistic effect of maternal AE coupled with adverse lifestyle factors.

### Transgenerational hyperglycemic effect of prenatal AE is attributed to pancreatic β-cell dysfunction in male offspring

**Fig. 3:**
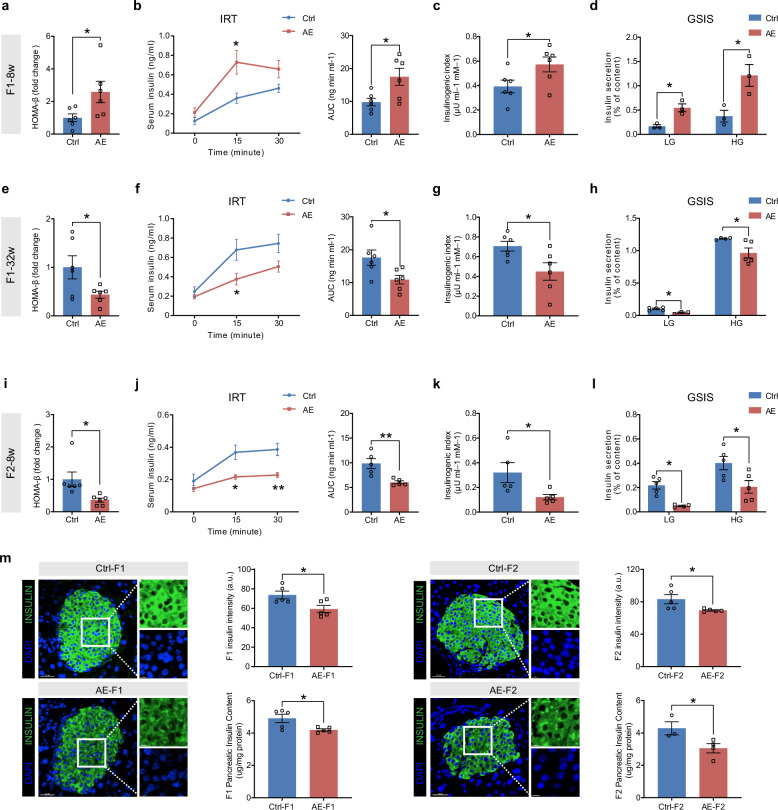
Prenatal AE causes transgenerational defects in insulin secretion of male offspring. **a** HOMA-β levels of F1 male offspring mice at 8 weeks of age (*n* = 6). **b** Serum insulin levels at 0, 15, and 30 min during insulin release test (IRT) after intraperitoneal glucose injection and the corresponding AUC in F1 male offspring at 8 weeks of age (*n* = 6). **c** Insulinogenic index during the IRT of F1 male offspring at 8 weeks of age (*n* = 6). **d** Islets isolated from Ctrl- and AE-F1 male offspring at 8 weeks of age were stimulated with 3.3 mM or 16.7 mM glucose and insulin secretion was assayed (*n* = 3). **e** HOMA-β levels of F1 male offspring mice at 32 weeks of age (*n* = 6). **f** Serum insulin levels at 0, 15, and 30 min of IRT after intraperitoneal glucose injection and the corresponding AUC in F1 male offspring at 32 weeks of age (*n* = 6). **g** Insulinogenic index during the IRT of F1 male offspring at 32 weeks of age (*n* = 6). **h** Islets isolated from Ctrl- and AE-F1 male offspring at 32 weeks of age were stimulated with 3.3 mM (Ctrl: *n* = 5; AE: *n* = 3) or 16.7 mM (Ctrl: *n* = 4; AE: *n* = 5) glucose and insulin secretion was assayed. **i** HOMA-β levels of F2 male offspring mice at 8 weeks of age (*n* = 6). **j** Serum insulin levels at 0, 15, and 30 min of IRT after intraperitoneal glucose injection and the corresponding AUC in F2 male offspring at 8 weeks of age (*n* = 5). **k** Insulinogenic index during the IRT of F2 male offspring at 8 weeks of age (*n* = 5). **l** Islets isolated from Ctrl- and AE-F2 male offspring at 8 weeks of age were stimulated with 3.3 mM (Ctrl: *n* = 5; AE: *n* = 4) or 16.7 mM (Ctrl: *n* = 5; AE: *n* = 5) glucose and insulin secretion was assayed. **m** Representative pancreatic sections were stained for INSULIN (green) and DAPI (blue), and insulin intensities, as well as pancreatic insulin contents, were quantified from 32-week-old F1 mice and 8-week-old F2 mice (*n* = 3‒5). Scale bars, 30 μm. Data are presented as mean ± SEM. *P < 0.05, **P < 0.01; significance is assessed by two-tailed unpaired Student’s *t*-test.

On the basis of the above findings, we next explored the underlying mechanisms that could explain the hyperglycemic phenotypes in AE male offspring. Whole-body glucose homeostasis is maintained by orchestrated insulin secretion from pancreatic β-cells^23,24^. Indeed, at 8 weeks, AE-F1 males exhibited a significant increase in HOMA-β levels (Fig. 3a), which closely mirrors the phenotype of cohort sons. These mice also showed remarkably increased serum insulin levels following intraperitoneal glucose administration (Fig. 3b), accompanied by a substantial increase in the insulinogenic index (Fig. 3c), suggesting an early-life hyperfunctioning of β-cells. Consistently, ex vivo glucose-stimulated insulin secretion (GSIS) assays with primary islets from F1 mice revealed significantly enhanced insulin secretion under both low- and high-glucose stimulations in the AE group (Fig. 3d). These results from AE-F1 mice are in accordance with higher HOMA-β observed in sons of hyperandrogenism women (Fig. 1b), indicating abnormally heightened β-cell function present at a young age after prenatal AE.

By 32 weeks of age, HOMA-β levels, insulin release in response to glucose, as well as the insulinogenic index were all markedly declined in AE-F1 mice (Fig. 3e‒g). GSIS further revealed a significant reduction in insulin secretion from the islets of these older AE-F1 mice compared to controls (Fig. 3h). This decline in β-cell function aligns closely with the observed progressive deterioration of glucose tolerance as the mice age (Fig. 2c). In the F2 generation, AE males displayed markedly decreased HOMA-β levels, insulin release, and significantly impaired GSIS from islets as early as 8 weeks (Fig. 3i‒l), without substantial differences in insulin sensitivity between the two groups (Supplementary Fig. S3a‒c). Moreover, pancreatic immunofluorescence and insulin content measurement revealed a noticeable reduction in insulin levels of both AE-F1 and AE-F2 islets, suggesting reduced insulin synthesis in β-cells of these mice (Fig. 3m). Collectively, these data demonstrate that the transgenerational diabetes susceptibility in AE offspring is primarily due to compromised pancreatic β-cell function.

### Prenatal AE alters DNA methylations in sperm that associate with insulin secretion

**Fig. 4:**
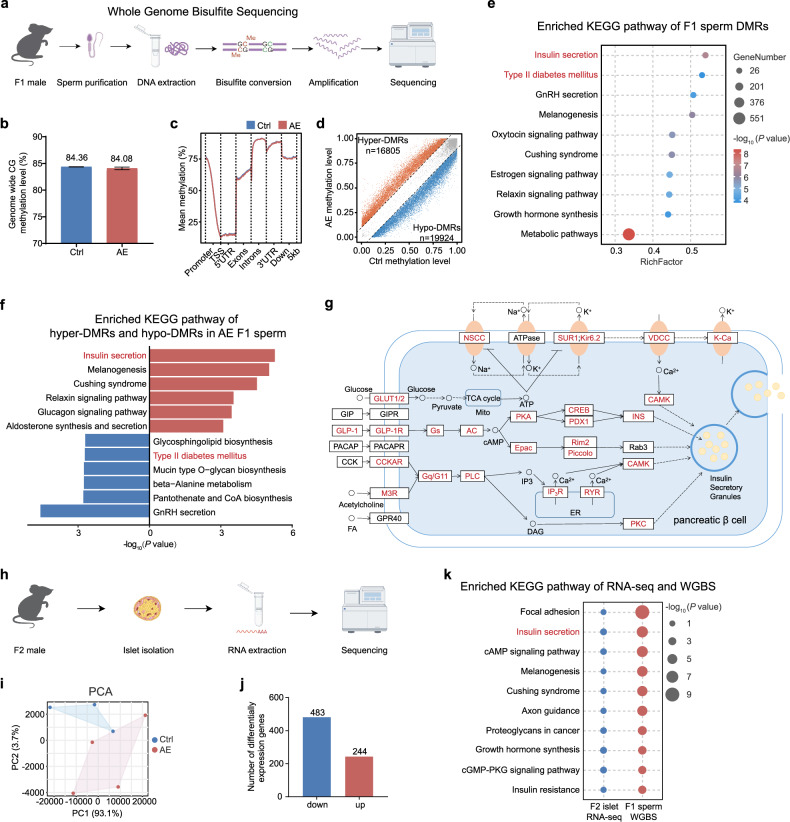
DNA methylation signatures in F1 sperm and transcriptomic alterations in F2 islets. **a** Schematic diagram for WGBS of sperms from F1 male mice. **b** Genome-wide CG methylation levels of Ctrl- and AE-F1 sperms. **c** Metaplot of CG methylation levels of Ctrl- and AE-F1 sperms. **d** Methylome comparison between Ctrl- and AE-F1 sperms using DMRs. **e** Top 10 enriched metabolic pathways from KEGG analysis of genes with DMRs in F1 sperm. **f** Top 5 enriched metabolic pathways from KEGG analysis of genes with hyper-DMRs (red) and hypo-DMRs (blue) in F1 sperm. **g** Red represents genes with DMRs identified by sperm WGBS in the insulin secretion pathway. **h** Schematic diagram illustrating the experimental approach used for RNA sequencing of F2 islets. **i** PCA of transcriptome from control (*n* = 3) and AE-F2 islets (*n* = 4). **j** Number of differentially upregulated and downregulated genes in AE-F2 islets compared with controls. **k** Top 10 enriched KEGG pathway shared between F2 islet transcriptome and F1 sperm methylome.

DNA methylation is proposed to be one of the major epigenetic inheritance mechanisms that mediate the transmission of paternal metabolic traits in sperm^25–27^. To determine the potential role of aberrant DNA methylation in the AE male germline-dependent transmission of hyperglycemic traits, F1 sperms from both control and AE males were subjected to whole-genome bisulfite sequencing (WGBS), and then the methylation alterations were assessed (Fig. 4a). Despite no significant differences in global CG methylation levels or patterns between the two groups (Fig. 4b, c), we identified 16,805 hypermethylated and 19,924 hypomethylated differentially methylated regions (DMRs) (Fig. 4d), which predominantly located within intergenic regions, introns, CpG islands, and exons (Supplementary Fig. S4a). These DMRs correspond to 5179 exclusively hypermethylated genes and 5222 hypomethylated genes (Supplementary Fig. S4b), pointing to the pronounced methylomic changes resulting from prenatal AE.

To gain insights into the functions of DMR-associated genes, we conducted pathway enrichment analyses to pinpoint the implicated biological processes. Analysis of the Kyoto Encyclopedia of Genes and Genomes (KEGG) pathway highlighted significant enrichments of several metabolic pathways, among which insulin secretion and T2D mellitus were listed at the top (Fig. 4e), aligning with the hyperglycemia and β-cell dysfunction observed in AE males. Of note, genes exhibiting hypermethylation showed strong enrichment in the insulin secretion pathway, whereas hypomethylated genes were significantly enriched in T2D mellitus (Fig. 4f). Remarkably, the vast majority of genes within the insulin secretion pathway were differentially altered in methylation (Fig. 4g). Further, the analysis of differentially methylated cytosines (DMCs) identified 36,341 hypermethylated and 38,214 hypomethylated DMCs in sperm WGBS (Supplementary Fig. S4c). Subsequent KEGG pathway analysis corroborated the enrichments in insulin secretion and T2D mellitus pathways for these DMC-associated genes (Supplementary Fig. S4d).

To further understand the molecular mechanism through which sperm methylation changes influence offspring glucose metabolism, we performed transcriptomic RNA sequencing (RNA-seq) analysis of islets from control-F2 and AE-F2 males (Fig. 4h), which were derived from F1 sperms. Principle component analysis (PCA) showed that the global transcriptome could be clearly discriminated between control and AE islets (Fig. 4i). Analysis of differentially expressed genes (DEGs) identified that 244 genes were upregulated, whereas 483 genes were downregulated in AE-F2 islets compared with controls (Fig. 4j). Integrated pathway analysis, combining WGBS data from F1 sperms and RNA-seq from F2 islets, pinpointed insulin secretion as the most prominently enriched metabolic pathway shared between the two datasets (Fig. 4k). These findings suggest that differentially methylated genes in AE-F1 sperm, especially those implicated in insulin secretion, are critical for the epigenetic inheritance of hyperglycemic phenotypes.

### DNA methylation changes persisting from AE-F1 sperm to AE-F2 islets contribute to suppressed expression of β-cell functional genes

**Fig. 5:**
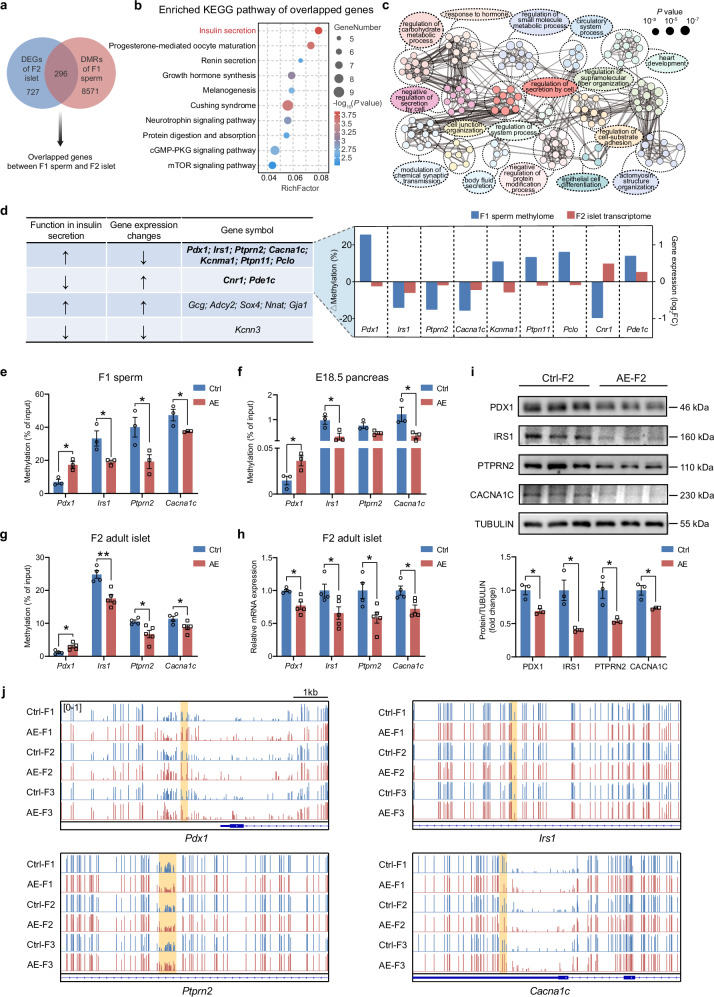
Aberrant DNA methylations persisted from AE-F1 sperm to AE-F2 islets contribute to abnormal expression of β-cell functional genes. **a** Venn diagram of the 727 DEGs in F2 islet and the 8571 genes with DMRs in F1 sperm, with 296 genes overlapping between the two conditions. **b** Top 10 pathways identified by KEGG enrichment analysis of the 296 overlapped genes in **a**. **c** Network analysis of the enriched pathways for 296 overlapped genes commonly altered between the WGBS and the RNA-seq datasets. **d** List of the genes in the insulin secretion pathway identified in **b** and **c**. It details the gene functions, differential methylation levels of insulin secretion genes in AE-F1 sperm, and gene expression changes in AE-F2 islet. **e** MeDIP-qPCR analyses for DNA methylation levels of *Pdx1*, *Irs1*, *Ptprn2*, and *Cacna1c* in Ctrl-F1 and AE-F1 sperms (*n* = 3). **f** MeDIP-qPCR analyses for DNA methylation levels of *Pdx1*, *Irs1*, *Ptprn2*, and *Cacna1c* in Ctrl-F2 and AE-F2 E18.5 pancreases (*n* = 3). **g** MeDIP-qPCR analyses for DNA methylation levels of *Pdx1*, *Irs1*, *Ptprn2*, and *Cacna1c* in Ctrl-F2 (*n* = 4) and AE-F2 adult islets (*n* = 5). **h** qRT-PCR analyses for mRNA expression levels of *Pdx1*, *Irs1*, *Ptprn2*, and *Cacna1c* in Ctrl-F2 (*n* = 4) and AE-F2 adult islets (*n* = 5). **i** Protein levels of PDX1, IRS1, PTPRN2, and CACNA1C were determined by western blot assay in islets from Ctrl-F2 and AE-F2 adult mice. **j** Methylation profile of the insulin secretion genes in F1‒F3 sperms. Vertical bars above the horizontal line indicate the methylation level (0‒1) at individual CpG site. The box indicates a DMR in the promoter or gene-body region. Data are presented as mean ± SEM. *P < 0.05, **P < 0.01; significance is assessed by two-tailed unpaired Student’s *t*-test.

To analyze whether DNA methylation changes in AE-F1 sperm might affect gene expression in the offspring, we searched for overlaps between DMR-associated genes in F1 sperm and DEGs in F2 islets. This approach uncovered 296 overlapped genes that are significantly altered in both F1 sperm methylome and F2 islet transcriptome (Fig. 5a), potentially mediating the transgenerational transmission of phenotypes. To be noted, over 40% of DEGs (296 out of 727) in islets also exhibited differential methylations in F1 sperms (Fig. 5a), indicating that alterations in transcription might be partly driven by methylation changes. Enrichment analysis of these overlapped genes further confirmed insulin secretion as the most affected pathway (Fig. 5b). These overlapped genes were also enriched in core gene ontology terms related to cell secretion (Fig. 5c). Through these analyses, a number of genes involved in insulin secretion with both differential methylations and transcriptions were identified (Fig. 5d). Notably, among the insulinotropic genes*, Pdx1*, *Irs1*, *Ptprn2*, *Kcnma1*, *Cacna1c, Ptpn11*, and *Pclo* were selectively downregulated in AE-F2 islets, which function as essential transcription factors or stimulation-secretion coupling factors for β-cells^28–32^. Additionally, *Cnr1* and *Pde1c* functioning in repressing insulin secretion^33,34^ were significantly upregulated in AE-F2 islets (Fig. 5d).

To explore whether inheritance of the observed phenotypes was attributed to DNA methylation changes of these β-cell functional genes, we examined the DMRs of these key genes in offspring islets by performing methylated DNA immunoprecipitation (MeDIP)-qPCR (Supplementary Fig. S5a). MeDIP-qPCR analysis verified the significant DNA methylation aberrations of these key genes in AE-F1 sperm (Fig. 5e; Supplementary Fig. S5b). Notably, most of these DMRs in insulin secretion genes showed significantly consistent trends of changes in both fetal mouse pancreas on embryonic day E18.5 (Fig. 5f; Supplementary Fig. S5c) and adult F2 islets (Fig. 5g; Supplementary Fig. S5d), indicating persistence of the DNA methylation marks throughout offspring development. Specifically, significant hypermethylation in the promoter region of *Pdx1*, which encodes a transcription factor vital for insulin synthesis and secretion^28^, was identified in AE groups of F1 sperm, E18.5 pancreas, and F2 adult islets (Fig. 5e‒g). Additionally, hypomethylations within the introns of *Irs1* and *Ptprn2* as well as in the CpG islands of *Cacna1c* were revealed in AE groups. Moreover, mRNA and protein expression levels of PDX1, IRS1, PTPRN2, and CACNA1C were markedly reduced in AE-F2 islets (Fig. 5h, i), which is consistent with the notion that promoter methylation inhibits transcription and gene body methylation typically increases transcription^35,36^. These results collectively suggest that the altered DNA methylations in insulin secretion genes originate from AE-F1 sperm and are transmitted during embryo development into AE-F2 islets, which in turn suppress gene expressions essential for β-cell function, leading to glucose intolerance in the offspring.

We further traced the methylation patterns of these key β-cell functional genes in sperms across F1 to F3 generations. Analysis of the WGBS data revealed that AE-F2 sperm retained differential DNA methylations in key β-cell functional genes, including *Pdx1*, *Irs1, Ptprn2, Cacna1c*, mirroring the methylation changes initially acquired in AE-F1 sperm (Fig. 5j). However, in AE-F3 sperm, these DNA methylation differences in β-cell functional genes were no longer present (Fig. 5j). This pattern of methylation changes aligns well with the transmission of metabolic phenotypes, where glucose intolerance was present in AE-F3 mice but lost in AE-F4 mice. These findings suggest that the acquired DNA methylation signatures persisted from F1 to F2 generation but were subsequently erased in F3 sperm.

### Shared methylation signatures in AE-F1 sperm and T2D patients are validated in blood of AE-sons

**Fig. 6:**
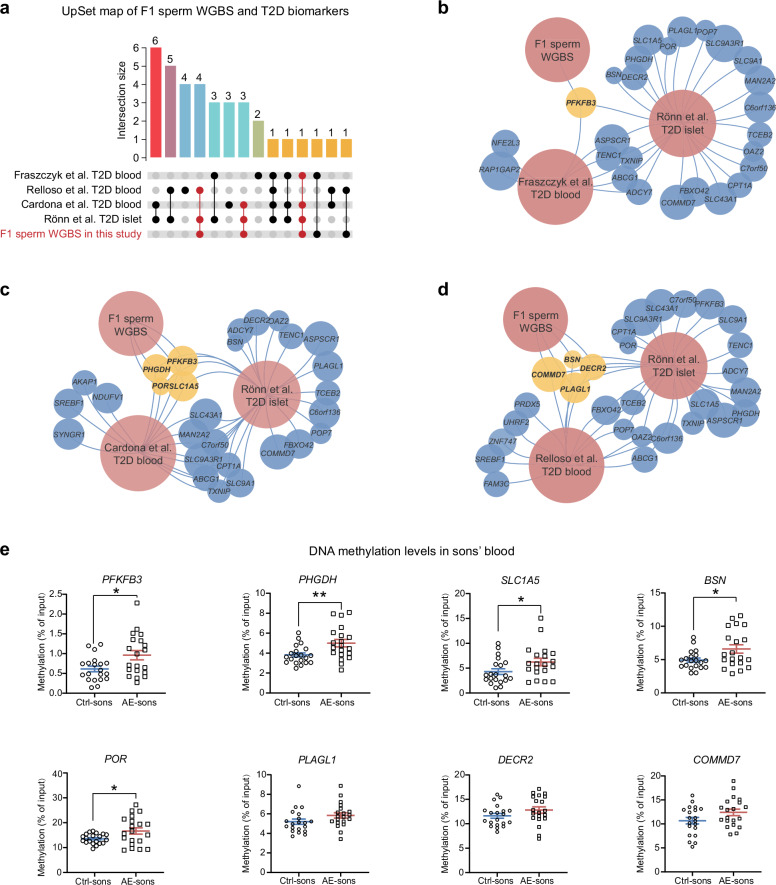
DNA methylation alterations shared by AE-F1 sperm, T2D patients, and blood of sons with maternal hyperandrogenism. **a** UpSet map illustrating gene numbers intersected by sperm WGBS and published DNA methylation data from T2D subjects. **b** Venn network of F1 sperm WGBS, T2D islets, and T2D blood study performed by Fraszczyk et al.^40^ showing common methylated genes. **c** Venn network of F1 sperm WGBS, T2D islets, and T2D blood study performed by Cardona et al.^37^ showing common methylated genes. **d** Venn network of F1 sperm WGBS, T2D islets, and T2D blood study performed by Relloso et al.^39^ showing common methylated genes. **e** MeDIP-qPCR analyses for DNA methylation levels in blood of control sons (*n* = 20) and sons with maternal hyperandrogenism (*n* = 20). Data are presented as mean ± SEM. *P < 0.05, **P < 0.01; significance is assessed by two-tailed unpaired Student’s *t*-test.

Epigenetic signatures prospectively associated with T2D incidence serve as powerful biomarkers for early detection of high-risk individuals^37^. To explore whether DNA methylation alterations in AE offspring could indicate future T2D risk in humans, we compared differentially methylated genes in AE-F1 sperm with documented DNA methylation changes in the islets of T2D patients, as well as in the blood of individuals prior to T2D diagnosis^37–40^ (Fig. 6a). This comparative analysis identified eight genes with differential methylation alterations shared across mouse AE-F1 sperm, human T2D islets, and pre-diagnostic T2D blood (e.g. *PFKFB3, PHGDH*, *PLAGL1*, *SLC1A5*, *BSN*, *POR*, *DECR2*, and *COMMD7*) (Fig. 6b‒d). It is worth noting that these genes are also implicated in β-cell function regulation or adult-onset diabetes^41–44^, underscoring their potential role in T2D progression.

To validate the identified methylation markers, we assessed their levels in sons from hyperandrogenism mothers, who were still at the child stage but exhibited altered β-cell function. The above eight genes with common alterations between mouse F1 sperm and human T2D subjects were selected for MeDIP-qPCR analysis using blood samples from sons in our birth cohort (Supplementary Fig. S5e). Remarkably, five out of these eight methylation markers showed significant alterations in the blood of AE-sons, which are *PFKFB3, PHGDH*, *SLC1A5*, *BSN*, and *POR* (Fig. 6e). These changes in methylation patterns were consistent with those observed in AE-F1 sperm. Collectively, these findings suggest that the identified methylation signatures could serve as early biomarkers to screen children at high metabolic risk, potentially forecasting future T2D susceptibility.

### CR and Met ameliorate metabolic dysfunctions in AE-F1 males and block their transmission to offspring

**Fig. 7:**
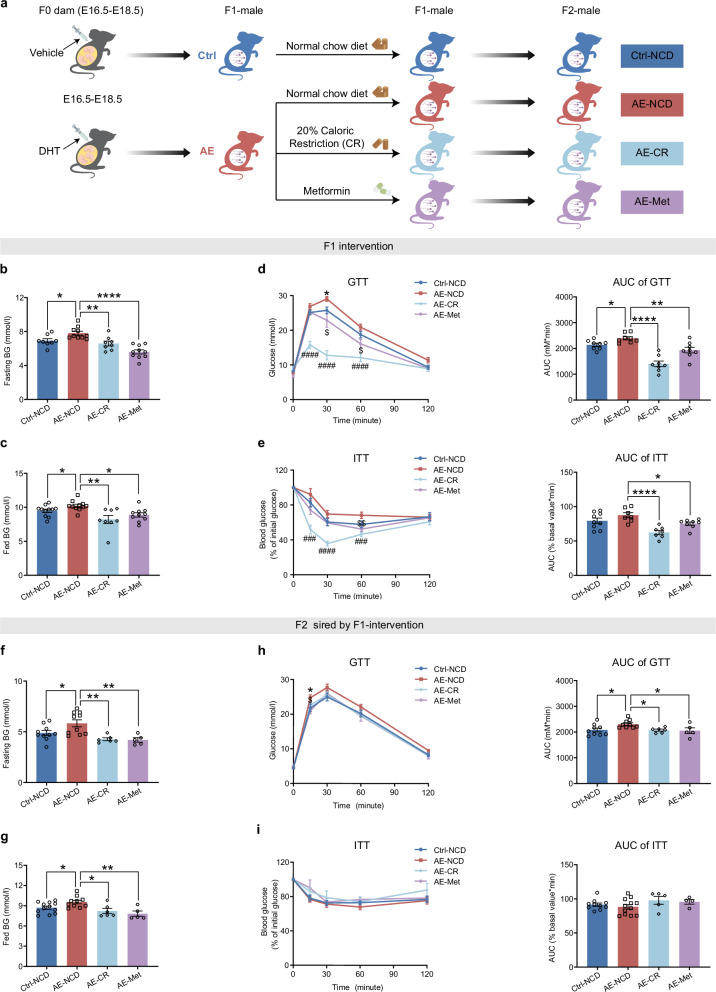
CR and Met ameliorate metabolic dysfunctions in AE-F1 mice and block their transmission to offspring. **a** Schematic illustration of CR and Met interventions for F1 male offspring. Mice fed ad libitum NCD were used as controls. **b** Blood glucose levels upon 6 h of fasting in F1 males after the indicated interventions (Ctrl-NCD, *n* = 8; AE-NCD, *n* = 10; AE-CR, *n* = 8; AE-Met, *n* = 10). **c** Fed blood glucose levels in F1 males after the indicated interventions (Ctrl-NCD, *n* = 11; AE-NCD, *n* = 11; AE-CR, *n* = 8; AE-Met, *n* = 10). **d** IPGTT and the related AUC of F1 males after the indicated interventions (*n* = 8). **e** ITT and the related AUC of F1 males after the indicated interventions (Ctrl-NCD, *n* = 9; AE-NCD, *n* = 7; AE-CR, *n* = 7; AE-Met, *n* = 8). **f** Blood glucose levels upon 6 h of fasting in F2 male offspring born from intervened F1 males as indicated (Ctrl-NCD, *n* = 10; AE-NCD, *n* = 10; AE-CR, *n* = 6; AE-Met, *n* = 5). **g** Fed blood glucose levels in F2 male offspring born from intervened F1 males as indicated (Ctrl-NCD, *n* = 12; AE-NCD, *n* = 10; AE-CR, *n* = 6; AE-Met, *n* = 5). **h** GTT and the related AUC of F2 male offspring born from intervened F1 males as indicated (Ctrl-NCD, *n* = 9; AE-NCD, *n* = 9; AE-CR, *n* = 6; AE-Met, *n* = 5). **i** ITT and the related AUC of F2 male offspring born from intervened F1 males as indicated (Ctrl-NCD, *n* = 10; AE-NCD, *n* = 12; AE-CR, *n* = 5; AE-Met, *n* = 4). Data are presented as mean ± SEM. *, Ctrl-NCD vs AE-NCD; statistical analyses of **b, c, d, e, f, g, h**, and **i** are performed using two-tailed unpaired Student’s *t*-test. #, AE-NCD vs AE-CR; $, AE-NCD vs AE-Met; statistical analyses of **b, c, d, e, f, g, h**, and **i** are performed using one-way ANOVA with Tukey’s multiple comparison test. *P < 0.05, $*P* < 0.05, **P < 0.01, $$*P* < 0.01, ###*P* < 0.001, ****P < 0.0001, ####*P* < 0.0001.

Recent studies have demonstrated CR’s potential in β-cell recovery and T2D remission^45,46^, while Met is renowned for its β-cell protective effects in glycemic control^47^. Since both CR and Met could target insulin secretion dysfunction, which is the core pathogenic mechanism in AE male offspring, we explored their therapeutic potentials in alleviating transgenerational hyperglycemic inheritance. In our study, CR was performed by feeding mice with 80% of their normal consumption starting at 16 weeks of age (Supplementary Fig. S6a), when AE-F1 males already developed glucose intolerance. Simultaneously, another group of AE-F1 mice were treated with Met at a dosage of 250 mg/kg daily for four weeks^48,49^ (Fig. 7a).

Following the interventions, both AE-CR and AE-Met groups exhibited significant body weight reductions compared to the NCD-fed AE controls, showing an average weight reduction of 7.6% and 2.0%, respectively (Supplementary Fig. S6b, c). More importantly, both fasting and fed blood glucose levels were significantly decreased in AE-CR and AE-Met mice compared with that of AE-NCD mice (Fig. 7b, c). Furthermore, AE-CR and AE-Met mice displayed significantly improved glucose tolerance as well as markedly enhanced insulin sensitivity (Fig. 7d, e). These results confirm the efficacy of CR and Met in mitigating the hyperglycemia induced by prenatal AE.

To examine the effects of these paternal interventions on subsequent generations, F1 mice subjected to CR or Met were mated with normal female mice to produce F2 male offspring, which were raised without any interventions (Fig. 7a). Body weight and food intake across the F2 groups were similar (Supplementary Fig. S6d, e). Remarkably, CR and Met interventions restored the aberrantly heightened fasting and fed blood glucose in AE-F2 males to the levels of NCD controls (Fig. 7f, g). Moreover, paternal CR or Met treatment normalized the glucose intolerance in AE-F2 males, as evidenced by significantly decreased blood glucose levels during IPGTT (Fig. 7h), while insulin sensitivity remained comparable (Fig. 7i). Collectively, the hyperglycemic defects in AE male offspring could be corrected by CR or Met not only in the directly treated F1 generation but also in their F2 offspring.

### CR and Met restore the aberrant DNA methylation and expression of β-cell functional genes in AE offspring mice

**Fig. 8:**
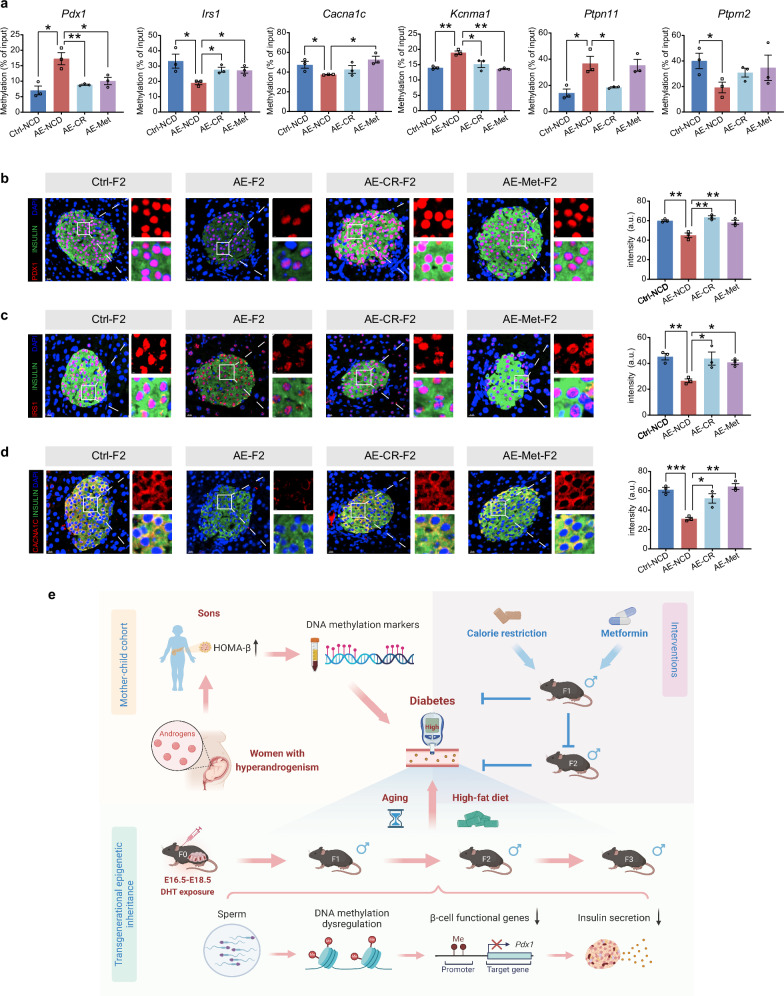
CR and Met restored the aberrant DNA methylation and expression of β-cell functional genes in AE offspring mice. **a** MeDIP-qPCR analyses for DNA methylation levels of *Pdx1*, *Irs1*, *kcnma1*, *Ptprn2*, *Cacna1c*, and *Ptpn11* in sperms from Ctrl, AE, AE-CR, and AE-Met F1 male mice (*n* = 3). **b** Representative pancreatic sections were stained for PDX1 (red), INSULIN (green), and DAPI (blue) from paternal Ctrl, AE, AE-CR, and AE-Met treated F2 male mice. PDX1 fluorescent intensities in insulin-positive β-cells were quantified (*n* = 3). Scale bars, 50 μm. **c** Representative pancreatic sections were stained for IRS1 (red), INSULIN (green), and DAPI (blue) from paternal Ctrl, AE, AE-CR, and AE-Met treated F2 male mice. IRS1 fluorescent intensities in insulin-positive β-cells were quantified (*n* = 3). Scale bars, 50 μm. **d** Representative pancreatic sections were stained for CACNA1C (red), INSULIN (green), and DAPI (blue) from paternal Ctrl, AE, AE-CR, and AE-Met treated F2 male mice. CACNA1C fluorescent intensities in insulin-positive β-cells were quantified (*n* = 3). Scale bars, 50 μm. **e** Schematic working model: maternal AE predisposes the sons to β-cell dysfunction and DNA methylation changes. In mice, prenatal AE induces DNA methylation reprogramming via sperm and defective insulin secretion of offspring islets, resulting in hyperglycemia and glucose intolerance in F1‒F3 male progeny with aging and high-fat diet. CR and Met interventions restore glucose homeostasis and prevent the transmission of hyperglycemia to offspring. Image was created with BioRender.com. Data are presented as mean ± SEM. *P < 0.05, **P < 0.01, ***P < 0.001. Statistical analyses of **a, b, c**, and **d** for Ctrl-NCD vs AE-NCD are performed using two-tailed unpaired Student’s *t*-test; statistical analyses for other groups excluding Ctrl-NCD are performed using one-way ANOVA with Tukey’s multiple comparison test.

To explore whether the therapeutic effects of CR and Met could be attributed to DNA methylation changes, we collected sperms from F1 control, AE, AE-CR, and AE-Met mice at the end of the treatment period and performed MeDIP-qPCR analyses. Among the key insulin secretion genes with concordant alterations of methylation in F1 sperm and alterations of transcription in F2 islet (Fig. 5d), DNA methylation levels of *Pdx1*, *Irs1*, and *Kcnma1* in the sperm of AE mice were significantly restored to normal conditions by both CR and Met treatments, whereas the restorations of *Ptprn2*, *Cnr1*, *Pclo*, and *Pde1c* followed a similar pattern although did not reach statistical significance (Fig. 8a; Supplementary Fig. S6f). Additionally, the aberrant methylation of *Cacna1c* was effectively normalized only by Met treatment, while the methylation of *Ptpn11* was corrected to control levels only by CR (Fig. 8a).

We further selected three β-cell functional genes that were found to be both differentially methylated and expressed in AE-F2 islets, *Pdx1*, *Irs1*, and *Cacna1c*, and assessed their protein expression levels after paternal interventions. In consistent with the down-regulated mRNA levels (Fig. 5h), immunofluorescent staining confirmed that PDX1, IRS1, and CACNA1C exhibited significantly decreased protein levels in the islet β-cells of AE-F2 mice compared to controls (Fig. 8b‒d). To be noted, the protein levels of these critical β-cell functional genes were completely restored to normal in F2 islets following paternal CR and Met treatments (Fig. 8b‒d). Taken together, the above results suggest that CR and Met can block the transmission of β-cell dysfunction to offspring by restoring DNA methylation patterns in androgen-exposed males, offering promising strategies to prevent the inheritance of diabetes risk to future generations.

## Discussion

In the present study, we demonstrate that maternal AE can be a inter- and transgenerational determinant of the offspring’s β-cell function, rendering them more vulnerable to diabetes (Fig. 8e). Transgenerational mice studies revealed that F1‒F3 male progeny from prenatal AE lineage develop hyperglycemia and glucose intolerance with advanced age and HFD, primarily due to defective insulin secretion. DNA methylation changes in F1 sperm and F2 islet underlie this transgenerational inheritance of impaired β-cell function. Shared methylation changes were also identified among AE mice, T2D patients, and blood of AE-sons, suggesting potential biomarkers for T2D risk. Furthermore, CR and Met interventions could effectively prevent the transmission of hyperglycemia.

Diabetes has become a global pandemic threatening the health of nearly half a billion people worldwide. The DOHaD theory posits that epigenetic alterations during early life can affect gene expression, disrupt signaling cascades, and alter disease susceptibility. Previous studies have established the embryonic developmental origins of diabetes, indicating that maternal adverse exposures, either pregestational or in utero, can lead to offspring glucose intolerance^26,50,51^. Employing both clinical mother-child cohort and transgenerational mouse model, our findings substantially advance the understanding of diabetes etiology by providing robust evidence that maternal AE is a triggering factor for offspring β-cell dysfunction and conveys a transgenerational T2D risk. Without action, this maternal hormone perturbation during critical developmental window might continue to fuel the T2D epidemic globally^52^. Therefore, our findings underscore the urgent clinical need of monitoring and managing hyperandrogenic status in women of reproductive age to safeguard the metabolic health of future generations.

Our observations elucidate a complex and dynamic interplay of maternal AE with metabolic stressors such as aging and obesity in shaping the metabolic trajectories of male offspring. At an early stage, β-cell function was abnormally hyper-activated after prenatal AE, as evidenced by improved glucose tolerance and enhanced insulin release in 8-week-old AE-F1 mice. This phenotype could be due to an initial adaptation or compensation for the in-utero environment of the F1 offspring islets. Over time, however, this β-cell functional compensation is overwhelmed in the face of metabolic stresses such as aging or an unhealthy lifestyle of high-fat diet, leading to a progressive decline of insulin secretion and escalating into overt T2D in AE-F1 male offspring. This scenario illustrates how environmental factors play crucial roles in the determination of “susceptible” islets and T2D risk^53,54^. As such, maternal AE sets the stage for hyperglycemic susceptibility, while an adverse metabolic lifestyle of the offspring acts as a secondary strike triggering the onset of diabetes. Our study highlights the importance to guide a reasonable dietary habit and establish a favorable lifestyle, particularly in children and adolescents.

DNA methylation is a pivotal epigenetic modification in transgenerational inheritance. Acquired DNA methylation of sperm partly undergoes erasure postfertilization in early pre-implantation embryos and is subsequently reestablished in post-implantation embryos^25,55^. In our study, the identification of differential methylation of β-cell functional genes persisting from F1 sperm to embryonic pancreas and adult F2 islets suggests that these acquired methylation marks might evade epigenetic reprogramming during embryonic development. The involved methylation changes in AE sperm could either be maintained escaping erasure or reestablished by global de novo DNA methylation during post-implantation development, which were further transmitted to pancreatic islets. Specifically, we identified significant hypermethylation at the *Pdx1* promoter and reduced transcription of a subset of insulinotropic genes in AE-F2 islets. Since promoter methylation is inversely correlated with transcription, this hypermethylation-induced transcriptional repression could undermine the efficacy of these genes essential for β-cell performance. Therefore, inherited DNA methylation could orchestrate metabolic reprogramming of β-cell function, resulting in disturbed glucose homeostasis in the AE offspring. However, the acquired epigenetic marks that persist for a few generations may eventually be lost due to the natural reprogramming of the germline, as seen in our observation that DMRs related to β-cell dysfunction disappeared in F3 sperm. It is also interesting to note that AE-F4 males showed a tendency towards improved glucose tolerance compared to controls under HFD, which might be due to adaptive epigenetic changes that enhance their resilience to metabolic stress. This reflects the potential of reversible epigenetic mechanism for evolutionary adaptation to cope with environmental challenges.

In contrast with genetic mutations, epigenetic changes generally occur at high frequencies and are thus potent biomarkers for disease susceptibility^56^. Intriguingly, our study uncovered several methylation markers consistently altered in AE-F1 sperm, the blood of sons from hyperandrogenism mothers, and blood of individuals before T2D onset. Our findings strongly suggest that sperm could serve as a valuable source to develop epigenetic biomarkers for offspring exposed to adverse prenatal environments, highlighting the potential of sperm as a marker cell. These data from our study further support the findings by Rönn et al.^38^ that specific DNA methylation signatures could serve as early indicators for predicting T2D risk, which could enable screening in high-risk children and facilitate prompt interventions. Furthermore, alterations in these methylated genes may play critical roles in the pathogenesis of T2D in adults. For instance, the methylation marker gene *BSN*, identified in our study, was recently found to be associated with severe adult-onset obesity and T2D^44^. Future studies involving larger prospective cohorts are warranted to validate the diagnostic efficacy of these methylation markers and to explore their potential pathogenic mechanisms.

Current therapeutic strategies for metabolic disease inheritance are limited. Progress toward precision interventions that are efficient to prevent the inheritance of metabolic diseases has been hindered due to the lack of a clear mechanistic etiology. CR and Met are well-established interventions known to improve insulin secretion under diabetogenic conditions^47^, yet limited information is available regarding their benefits for offspring metabolism. In our study, paternal applications of CR and Met have been shown to effectively normalize hyperglycemia of AE-F1 mice and further block their transmission to AE-F2 offspring, supporting their potential clinical utility in high-risk populations to improve offspring metabolic health.

In our study, as both interventions are efficient in reversing the aberrant methylation changes in AE-F1 sperm, they may share common mechanisms in modulating methylation signals. This notion is supported by evidence showing that both CR and Met can activate AMP-activated protein kinase (AMPK)^57^, which is known to be an energy sensor in metabolic homeostasis and has previously been linked to the regulation of DNA methylation. Specifically, AMPK directly phosphorylates and stabilizes TET2, which is a key dioxygenase in catalyzing 5-methylcytosine to 5-hydroxymethylcytosine, thereby leading to DNA demethylation and epigenome reprogramming^58^. CR has also been shown to transcriptionally regulate the expression of *Dnmt3b*, *Tet2*, and *Tet3* in mouse liver^59^. Additionally, Met’s activation of AMPK elevates cellular α-ketoglutarate levels, which further supports TET-mediated DNA demethylation^60^. These combined effects suggest that CR and Met potentially reprogram DNA methylation landscape in sperm through regulating key methylation and demethylation enzymes, although the underlying mechanisms still need to be further elucidated in future research.

In summary, the present study demonstrates the driving role and epigenetic inheritance mechanism of maternal AE on transgenerational β-cell dysfunction and diabetes susceptibility. Our results provide feasible and robust intervention strategies for addressing offspring metabolic disorders associated with prenatal AE, shedding light on the promotion of the metabolic health in future generations.

## Materials and methods

### Human studies

This study was approved by the Institutional Review Board of the Center for Reproductive Medicine, Shandong University (Approval Number: IRB 2021-98) and was in accordance with the principles of the Declaration of Helsinki. The mother-child birth cohort was established at Center for Reproductive Medicine, Institute of Women, Children and Reproductive Health, Shandong University, China. Written informed consents were obtained from all participating mothers. Women with pregestational basal hormone levels, including testosterone tested were included in this study, which were measured at menstrual cycle day 3 by chemiluminescence immunoassays. Hyperandrogenism was defined as circulating total testosterone level ≥ 48.1 ng/dL, according to the assay kit’s instruction. Their children conceived by assisted reproductive technologies or intracytoplasmic sperm injection were born between 2014 and 2021, and were followed up until 2023. The inclusion criteria for the offspring were males who completed growth assessments and provided blood samples for the measurement of metabolic biochemical parameters. Offspring who did not undergo growth and metabolic assessments, lacked parental anthoropometric information, or had any chronic diseases were excluded from the study. For the final analysis, 561 sons born to women with hyperandrogenism were matched 1:2 by maternal and child age with 1122 control sons born to women without hyperandrogenism. The ages of the sons ranged from 2 to 12 years at the end of follow-up.

BMI was calculated using the equation: weight (kg)/height (m^2^). The blood samples of sons were collected after overnight fasting and were tested within 24 h. Fasting blood glucose (FBG) levels were detected using the oxidase method and fasting insulin (FINS) levels were measured by chemiluminescence immunoassays. The homeostasis model assessment of insulin resistance (HOMA-IR) was calculated as (FINS mIU/L × FBG mmol/L)/22.5. Homeostasis model assessment of pancreatic β-cell function (HOMA-β) was calculated as 20 × FINS mIU/L/(FBG: 3.5 mmol/L).

For the assessment of blood DNA methylation levels, morning fasting blood samples were obtained from the children. A total of 40 blood samples have been analyzed from 20 sons born to women with hyperandrogenism and 20 sons born to women without hyperandrogenism.

### Mouse modeling, breeding, and feeding schemes

The animal studies were approved by the Institutional Review Board of the Center for Reproductive Medicine, Shandong University (Approval Number: IRB 2021-98). Wild-type C57BL/6 J (8-week-old) female and male mice were purchased from GemPharmatech Co. (Jiangsu, China). Mice were allowed to acclimatize for 2 weeks and housed under specific pathogen-free conditions in a temperature-controlled room (22‒25 °C) with a 12-h light/dark cycle and free access to food and water. All mice were visually inspected every week during cage change. Standard chow diet (3.44 kcal/g with 12.95% kcal fat, Beijing KEAO XIElI FEED Co., China) was given to all mice during breeding, lactation and growth unless otherwise indicated. The sample size, sex, and age of the animals used is specified in the text and/or figure legends. All the in vivo experiments were run using a double-blind procedure. All animal experiments were carried out according to the rules and guidelines of the local animal ethics committee.

For prenatal androgen treatment, females were checked daily for post-copulatory plugs after mating, and a plug on the morning after mating was considered embryonic day 0.5 (E0.5). Timed-pregnant adult (3‒4 months) F0 dams were injected daily subcutaneously in the interscapular area from E16.5 to E18.5 with 100 μL of a solution comprising 10 µL benzyl benzoate (B6630, Sigma-Aldrich, USA) and 90 µL corn oil (C7030, Solarbio, China) for control group or 250 μg dihydrotestosterone (A8380, Sigma-Aldrich, USA) dissolved in a mixture of 10 µL benzyl benzoate and 90 µL corn oil for AE group. We selected this specific exposure window at late pregnancy as it occurs subsequent to the developmental stages of gonadal and genital tract differentiation in mice, thus precluding any potential morphogenetic effects that exogenous androgen might induce^61^. Subsequently, pregnant F0 dams delivered the F1 generation mice.

F1 male offspring were mated with F1 unrelated wild-type females to generate F2 offspring, F2 male offspring were mated with F2 unrelated wild-type females to generate F3 offspring, and F3 male offspring were mated with F3 unrelated wild-type females to generate F4 offspring. All male mice used for mating were at the ages of 8‒10 weeks. The generated F1‒F4 male offspring of control and prenatal AE groups were monitored for metabolic phenotypes until 32 weeks of age. For the HFD treatment, F1‒F4 male mice were randomly assigned to Ctrl-NCD, AE-NCD, Ctrl-HFD, and AE-HFD groups. Obesity was induced by feeding mice a high-fat diet (60 kcal% fat, Research Diets) for 12 weeks and were then subjected to phenotypic testing. Mice were maintained on HFD until the end of the study.

### CR and Met interventions

To determine the food quantity for CR interventions, AE-F1 mice were housed individually and fed ad libitum with standard chow diet. Food intake was measured every day over a period of one week. AE-CR mice were fed 80% of their normal food consumption for 4 weeks starting at 16 weeks of age. AE-NCD mice consumed an average food of ~3.35 g/day (approximately 11.52 kcal/day), and each mouse in the AE-CR group was fed ~2.7 g/day (approximately 9.29 kcal/day). Another group of AE-Met mice was administered Met (Sigma) at a dose of 250 mg/kg per day for 4 weeks. At the end of the interventions, these mice were mated with F1 unrelated wild-type females to generate F2 offspring. F2 offspring born from paternal treated mice were raised without any intervention.

### Assessment of body weight and energy metabolism

The body weight of mouse was recorded weekly. Whole body fat mass was measured in avertin-anesthetized mice using dual-energy X-ray absorptiometry. Indirect calorimetry was conducted by metabolic cages (CLAMS-16, Columbus, America), which allowed for the measurement of food intake, gas exchange, and spontaneous locomotor activity. Mice were kept individually in metabolic cages for three consecutive days; the first day being considered an adaptation period (not analyzed) and 24 h readings were used for analysis after the adaption period. Parameters included energy expenditure: indirect gas calorimetry and adjusted for total body mass, and respiratory exchange ratio: VCO_2_/VO_2_ as the calculated ratio between volumes of CO_2_ produced and O_2_ consumed.

### Measurement of blood glucose and insulin levels

Blood glucose levels were measured via tail vein blood using a portable glucometer (OneTouch Verio, Life scan). Fed blood glucose levels were measured at 10 am.

Insulin levels were determined in serum samples or pancreatic tissues homogenized in acid-ethanol solution by an Insulin ELISA kit (ALPCO, Salem, NH, USA). Pancreatic insulin content was normalized over protein concentration as determined by BCA assay.

### Glucose and insulin tolerance tests

For the IPGTT, mice were fasted overnight for 16 h and injected intraperitoneally with 20% glucose (2 g/kg body weight). Blood glucose levels were measured at 0, 15, 30, 60, and 120 min after glucose administration. AUCs were calculated as an index of whole glucose excursion in the blood after glucose loading.

For insulin tolerance test (ITT), mice were fasted for 6 h starting at 8 am and then injected with insulin at a dose of 0.75 IU/kg body weight. Blood glucose levels were measured at 0, 15, 30, 60, and 120 min after the intraperitoneal injections. Data for ITT are presented as the percentage of basal blood glucose level.

### Isolation of pancreatic islets

Mouse pancreatic islets were isolated by collagenase digestion and dense gradient centrifugation as described previously^24^. Briefly, a type XI collagenase solution (Sigma) was injected through the common bile duct. The perfused pancreas was dissected and incubated at 37 °C. Then intact islets and exocrine cells were separated via centrifugation and manually picked under the microscope. Islets were cultured in RPMI-1640 medium with 10% FBS, 100 U/mL penicillin and 100 μg/mL streptomycin.

### Insulin secretion assay

For in vivo insulin release test, mice that had fasted overnight were injected with glucose at a dose of 2 g/kg body weight, then blood samples were taken from the tail vein at 0, 15, and 30 min after glucose injection. The serum insulin levels were determined using Mouse Insulin ELISA kits (ALPCO, Salem, NH, USA). We calculated the insulinogenic index by dividing the increment in serum insulin (ΔIns0–30; in μU/mL) by the increment in plasma glucose (ΔGlu0–30; in mM) during the 0 to 30 min time periods of the insulin release test.

For ex vivo glucose-stimulated insulin secretion, isolated islets from control or androgen-exposed mice were cultured in RPMI-1640 medium. To stimulate insulin secretion, islets were pre-incubated in Krebs-Ringer bicarbonate (KRB) buffer containing 3.3 mM glucose. Batches of ten size-matched islets were then incubated in KRB buffer containing 3.3 or 16.7 mM glucose as indicated for 1 h at 37 °C. The supernatants were collected for the measurement of insulin secretion. Islets were then extracted with 0.18 N HCl in 70% ethanol to determine total insulin content. Insulin levels were measured using a Mouse Insulin ELISA kit and insulin secretion was normalized as a percentage of total insulin content.

### Immunofluorescence and histological staining

Pancreatic sections embedded in paraffin were deparaffinized, rehydrated, and incubated with guinea pig anti-insulin primary antibody (Dako) combined with rabbit anti-PDX1 primary antibody (Abcam), rabbit anti-IRS1 primary antibody (Proteintech), or rabbit anti-CACNA1C primary antibody (Sigma) overnight at 4 °C. These sections were further incubated with anti-guinea pig combined with anti-rabbit secondary antibodies (Invitrogen), and nuclei were counterstained with DAPI (Vectashield mounting medium with DAPI, Vector Laboratories, USA). Images were captured by the confocal laser scanning microscope (Andor Technology PLC, Andor Dragonfly 200). Measurements of fluorescence intensities in insulin-positive area were performed by ImageJ software (National Institutes of Health).

White and brown adipose tissue were immersion-fixed in 4% PFA solution and stored at 4 °C. Paraffin-embedded tissue was sectioned at a thickness of 5 μm and stained with hematoxylin and eosin. One to two representative images were taken per section with a light microscope at 20× magnification (Leica, Germany). The adipocyte size was quantified using Image J.

### Western blot assay

Islets were lysed using RIPA buffer (Beyotime) supplemented with protease inhibitor. Equal concentrations of proteins were diluted in loading buffer and denatured. Protein lysates were loaded into the 10% SDS polyacrylamide gel, subjected to electrophoresis. and transferred to a 0.45 μm PVDF membrane (Millipore). After blocking in 5% milk for 1 h at room temperature, the membrane was incubated overnight with rabbit anti-PDX1 primary antibody (Abcam), rabbit anti-IRS1 primary antibody (Proteintech), rabbit anti-PTPRN2 primary antibody (Invitrogen), rabbit anti-CACNA1C primary antibody (Sigma), or mouse anti-TUBULIN antibody (Proteintech) at 4 °C. The membrane was incubated with peroxidase-conjugated secondary antibody for 1 h at room temperature. Immunoreactive proteins were visualized using an imaging system (Bio-Rad). Protein band intensity was quantified using Image Lab (Bio-Rad).

### Isolation of motile spermatozoa of sperm

Mature sperms were collected from the dissected cauda epididymis of adult male mice. After dissection to eliminate blood vessels and fat, the cauda epididymis was punctured with a needle. Sperms were then transferred to a 2 mL round bottom tube overlaid with G-IVF medium (10136, Vitrolife) prewarmed to 37 °C. Sperms were then subjected to a swim-up assay at 37 °C and the supernatant containing motile spermatozoa was harvested after 1 h. Purified spermatozoa were washed with PBS and snap-frozen in liquid nitrogen.

### WGBS

Sperms collected from control and AE mice were incubated in lysis buffer (10 mM Tris-HCl, 100 mM NaCl, 1 mM EDTA, 0.3% SDS, 10 mM DTT) with proteinase K at 55 °C for 5 h and genomic DNAs (1 μg) were extracted using TIANamp Genomic DNA Kit (Tiangen, Cat#DP304-02). DNA concentration and integrity were assessed by NanoDrop spectrophotometer and agarose gel electrophoresis respectively. Then DNA libraries for Enzymatic Methylation sequencing were prepared. Briefly, genomic DNAs were fragmented into 100‒300 bp by Sonication (Covaris) and purified with MiniElute PCR Purification Kit (QIAGEN). Purified DNA was bisulfite converted using an EpiArt DNA Enzymatic Methylation Kit (Vazyme). The fragmented DNAs were end repaired and a single “A” nucleotide was added to the 3’ end of the blunt fragments. Then the genomic fragments were ligated to methylated sequencing adapters. Finally, the converted DNA fragments were PCR amplified and sequenced using Illumina HiSeqTM 2500.

### Analysis of WGBS data

All bisulfite sequencing reads underwent initial trimming to remove adaptors and low-quality bases using fastp software. The obtained clean reads were mapped to the mouse reference genome (Ensembl_release110) using BSMAP software (version: 2.90) by default. Then a custom Perl script was used to call methylated cytosines and the methylated cytosines were tested with the correction algorithm described in Lister et al.^62^. The methylation level was calculated based on methylated cytosine percentage in the whole genome, in each chromosome and in different regions of the genome for each sequence context (CG, CHG and CHH). To assess different methylation patterns in different genomic regions, the methylation profile at flanking 2 kb regions and genebody (or transposable elements) was plotted based on the average methylation levels for each window. Differential DNA methylation between the two samples at each locus was determined using Pearson’s chi-square test (χ2) in methylKit. To identify DMCs, the minimum read coverage to call a methylation status for a base was set to 4, the absolute value of the difference in methylation ratio ≥ 0.25, and *P* ≤ 0.01. To identify DMRs, the minimum read coverage to call a methylation status for a base was set to 4, numbers of GC in each window ≥ 5, the absolute value of the difference in methylation ratio ≥ 0.1, and *P* ≤ 0.01. Enrichment analyses were performed by the OmicShare tools and collected terms with GO Biological Processes and KEGG Pathway. Network enrichment analysis was performed by Metascape (http://metascape.org) and visualized using Cytoscape.

### RNA isolation and quantitative PCR

Total RNAs from isolated islets were extracted using AllPrep DNA/RNA/Protein Mini Kit (Qiagen, Hiden, Germany) according to the manufacturer’s protocol. Isolated RNAs were reverse transcribed using the Prime Script RT Kit with gDNA Eraser (Takara, Shiga, Japan). Real-time quantitative PCR was performed using SYBR Premix Ex Taq Kit (Takara, Shiga, Japan) on a Light Cycler 480 System (Roche, Basel, Switzerland). Results were normalized to *β-Actin* mRNA levels. The primer sequences were listed in Supplementary Table S3.

### RNA-sequencing

Total RNAs from control and AE-F2 islets were extracted. RNA quality was assessed on an Agilent 2100 Bioanalyzer (Agilent Technologies, Palo Alto, CA, USA) and checked using RNase free agarose gel electrophoresis. After total RNA was extracted, eukaryotic mRNA was enriched by Oligo (dT) beads. Then the enriched mRNA was fragmented into short fragments using fragmentation buffer and reversely transcribed into cDNA by using NEBNext Ultra RNA Library Prep Kit for Illumina (NEB#7530, New England Biolabs, Ipswich, MA, USA). The purified double-stranded cDNA fragments were end repaired, A base added, and ligated to Illumina sequencing adapters. The ligation reaction was purified with the AMPure XP Beads (1.0X) and PCR amplified. The resulting cDNA library was sequenced using Illumina Novaseq6000. Clean reads were filtered and then aligned to the mouse reference genome with HISAT2 software (version 2.2.4). Differentially expressed genes were identified with the *P* value < 0.05 between the two groups.

### MeDIP-qPCR analysis

MeDIP was performed using MagMeDIP kit (Diagenode) according to the manufacturer’s instructions. Briefly, genomic DNA from sperms or islets were extracted using AllPrep DNA/RNA/Protein Mini Kit (Qiagen), and blood DNA was extracted using the DNA Blood Mini Kit (Qiagen) according to the manufacturer’s instructions. A total of 1 μg DNA was sheared by sonication and immunoprecipitation was performed using an antibody anti-5mC (33D3 clone, Diagenode) and magnetic beads, following MagMeDIP kit settings. One-tenth of the DNA samples were set aside at 4 °C for input. To check the efficiency of the MeDIP experiment, spike-in controls in vitro, including unmethylated (unDNA) and methylated DNA (meDNA) from *A. thaliana* were used. After magnetic beads washes, methylated DNA was isolated using the DNA Isolation Buffer protocol according to the MagMeDIP kit recommendations. The efficiency of the immunoprecipitation was assessed by performing qPCR using primers for spike-in control. Methylation quantification was calculated from qPCR data and reported as the recovery% = 2^(Ct_10%input_-3.32-Ct_IP_) × 100%. The primer sequences were listed in Supplementary Table S3.

### Statistical analysis

In the cohort study, continuous data were presented as mean ± SD. Characteristics of mothers and sons were compared by the unpaired *t*-test. Linear regression was used in phenotypic results to adjust for the effect of maternal BMI. All analysis were performed using SPSS v27.0 software (SPSS Inc., Chicago, IL, USA).

The mouse study results were expressed as mean ± SEM for the indicated number of observations. A two-tailed unpaired Student’s *t*-test was performed to compare two groups. Comparisons among multiple groups were determined by ANOVA or a mixed-effects analysis model with Tukey’s multiple comparison test using Prism 8 (Graphpad Software, San Diego, CA). Differences with *P* < 0.05 were considered statistically significant.

## Supplementary information

Supplementary information

## Data Availability

Sequencing data generated in this study have been deposited in the Gene Expression Omnibus database under accession code GSE266699 (WGBS) and GSE266255 (RNA-seq).
