# Supplementary material for: Transgenerational inheritance of diabetes susceptibility in male offspring with maternal androgen exposure
Source: Cell Discov. 2025 Feb 12;11:14. doi: 10.1038/s41421-025-00769-1 (PMC11814079; doi:10.1038/s41421-025-00769-1)
Supplement: Supplementary file 1 — Supplementary information [file 41421_2025_769_MOESM1_ESM.pdf]

Supplementary Information

Transgenerational inheritance of diabetes susceptibility in male offspring with maternal androgen exposure

Supplementary Table S1. Maternal characteristics of women with and without hyperandrogenism.

	Women without HA	Women with HA	*P*
n	1122	561	
T (ng/dL)	23.417±9.021	60.887±38.226	0.0001***
Age (y)	30.118±3.994	30.188±3.765	0.732
BMI (kg/m^2)	22.815±3.258	23.6±3.744	0.0001***
FSH (IU/L)	7.128±5.305	6.773±2.267	0.13
LH (IU/L)	5.429±3.997	7.805±5.895	0.0001***
E2 (pg/mL)	42.519±75.101	50.003±55.726	0.037*
PRL (ng/mL)	18.58±25.032	18.86±11.322	0.801
TSH (uIU/mL)	2.698±3.525	2.578±1.962	0.46

Data are presented as mean ± SD. Significance is assessed by two-tailed unpaired Student's *t*-test. Abbreviations: HA, hyperandrogenism; T, testosterone; BMI, body mass index; FSH, follicle-stimulating hormone; LH, luteinizing hormone; E2, estradiol; PRL, prolactin; TSH, thyroid-stimulating hormone.

Supplementary Table S2. The clinical characteristics of sons born to women with or without hyperandrogenism.

	Ctrl-sons	HA-sons	*P*	*P*-adjusted
n	1122	561		
Age (y)	5.275±2.117	5.452±2.324	0.131	0.077
BMI (kg/m^2)	16.516±2.592	16.74±2.849	0.119	0.137
FBG (mmol/L)	5.025±0.436	5.023±0.453	0.939	0.903
FINS (mIU/L)	5.631±4.463	5.985±4.82	0.136	0.414
HOMA-IR	1.298±1.101	1.373±1.143	0.193	0.508
**HOMA-β**	**71.248±53.082**	**79.612±75.569**	**0.019***	**0.037***

Data are presented as mean ± SD. Significance is assessed by two-tailed unpaired Student's *t*-test. *P*-adj: *P* values are adjusted by maternal BMI. Indicators with statistically significant differences are expressed in bold ($P < 0.05$). Abbreviations: HA, hyperandrogenism; BMI, body mass index; FBG, fasting glucose; FINS, fasting insulin; HOMA-IR, homeostasis model for insulin resistance; HOMA-β, homeostasis model for β-cell function.

Supplementary Table S3. Sequences of the primers used for RT-qPCR and MeDIP-qPCR.

Sequences of the primers used for RT-qPCR		
**Gene**	**Species**	**Primer sequence (5' to 3')**
β-*Actin* _forward	*Mus musculus*	TGTTACCAACTGGGACGACA
β-*Actin* _ reverse	*Mus musculus*	GGGGTGTTGAAGGTCTCAAA
*Pdx1*_forward	*Mus musculus*	TTCCCGAATGGAACCGAGC
*Pdx1*_ reverse	*Mus musculus*	GCGTGAGCTTTGGTGGATT
*Irs1*_forward	*Mus musculus*	TCTACACCCGAGACGAACACT
*Irs1*_ reverse	*Mus musculus*	TGGGCCTTTGCCCGATTATG
*Ptprn2*_forward	*Mus musculus*	GAGGATGGCTTGTGTGGATCA
*Ptprn2*_reverse	*Mus musculus*	CGGAACCTTTTGACATCTTCCAA
*Cacna1c*_forward	*Mus musculus*	TCCCGAGCACATCCCTACTC
*Cacna1c*_reverse	*Mus musculus*	ACTGACGGTAGAGATGGTTGC

Sequences of the primers used for MeDIP-qPCR			
**Gene**	**Species**	**Primer sequence (5' to 3')**	**Methylation site**
*Pdx1*_forward	*Mus musculus*	CCAGACAAGAAACACGGTGA	Chr5:147205601-147205800
*Pdx1*_ reverse	*Mus musculus*	GCGCTGGCAAGGATAGACT	
*Irs1*_forward	*Mus musculus*	GCCTTCAATGTTTCCATCAATACC	Chr1:82221901-82222100
*Irs1*_ reverse	*Mus musculus*	AGCTCCCTGTATCCTTTTCATTCA	
*Ptprn2*_forward	*Mus musculus*	ATAAGTCTTCGGCGCTGCTC	Chr12:116560701-116561200
*Ptprn2*_reverse	*Mus musculus*	ATTTAGCCAGCGCCCGTATC	
*Cacna1c*_forward	*Mus musculus*	CTTACACCGGGGACACCTTT	Chr6:118569101-118569300
*Cacna1c*_reverse	*Mus musculus*	AGTGCTTCTTCTGTACGTGGT	
*Kcnma1*_forward	*Mus musculus*	TTTGCGCACACTTGGAAAGC	Chr14:23358301-23358600
*Kcnma1*_reverse	*Mus musculus*	TGGGCTGAGGTATCGCAAAA	
*Pclo*_forward	*Mus musculus*	TACCTCGCAGGTTACTGGGT	Chr5:14673301-14673600
Pclo _reverse	*Mus musculus*	CTCTTGCCCGACCTGACTG	
*Pde1c*_forward	*Mus musculus*	CCCTCTGTTGGAAAGGCACT	Chr6:56159001-56159200
*Pde1c*_reverse	*Mus musculus*	TGGGGCTATGGTTCTCTGTG	
*Cnr1*_forward	*Mus musculus*	GTTTGTCTACTGGGTCAGTGTGT	Chr4:33931301-33931500
*Cnr1*_reverse	*Mus musculus*	GGGAACCCCTCAATGTTTTGAC	
*Ptpn11*_forward	*Mus musculus*	CTCTGTCCCGTTCTCTGCTC	Chr5:121278401-121278600
*Ptpn11*_reverse	*Mus musculus*	GGGAAAGAGGCGGAGTGAAA	
*PFKFB3*_forward	*Homo sapiens*	AGAGTTGTGTGACTCCTGCG	chr10:6145842-6146049
*PFKFB3*_ reverse	*Homo sapiens*	GGAGTCTCGGGTGTTGAGTG	
*PHGDH*_forward	*Homo sapiens*	AGAGTTACAGGCGGAACAGC	chr1:119708470-119708810
PHGDH _ reverse	*Homo sapiens*	GGGAAAAGGTCCTAGTGCGT	
*SLC1A5*_forward	*Homo sapiens*	CCACATCCTCCATCTCCACG	chr19:46778792-46779015
*SLC1A5*_ reverse	*Homo sapiens*	GATGTGGGTAGGGTGCTGAG	
*BSN*_forward	*Homo sapiens*	CATCACAGAGCATAGTCCGC	chr3:49653374-49653573
*BSN*_ reverse	*Homo sapiens*	GGGCCACCATTTTGCTTCATT	
*POR*_forward	*Homo sapiens*	GAACTAAGCAGGGAGGAGGC	chr7:75987317-75987513
*POR*_ reverse	*Homo sapiens*	GTCTGTCTGTCCGTCCTCTG	
*PLAGL1*_forward	*Homo sapiens*	CTACATGCTTCCTCTCTTCTTTCAG	chr6:144057798-144057954
*PLAGL1*_ reverse	*Homo sapiens*	AGTCCATGAACCTGAGGGCTT	
*DECR2*_forward	*Homo sapiens*	AACCTGGCCTCTTTCTTAACAG	chr16:402227-402504
*DECR2*_ reverse	*Homo sapiens*	ATGAAGAGCTGCTGGGCAAA	
*COMMD7*_forward	*Homo sapiens*	TAGTCTCTCCCAACTCCCGC	chr20:32743239-32743360
*COMMD7*_ reverse	*Homo sapiens*	GACATGCAGCAGCTGAACC	

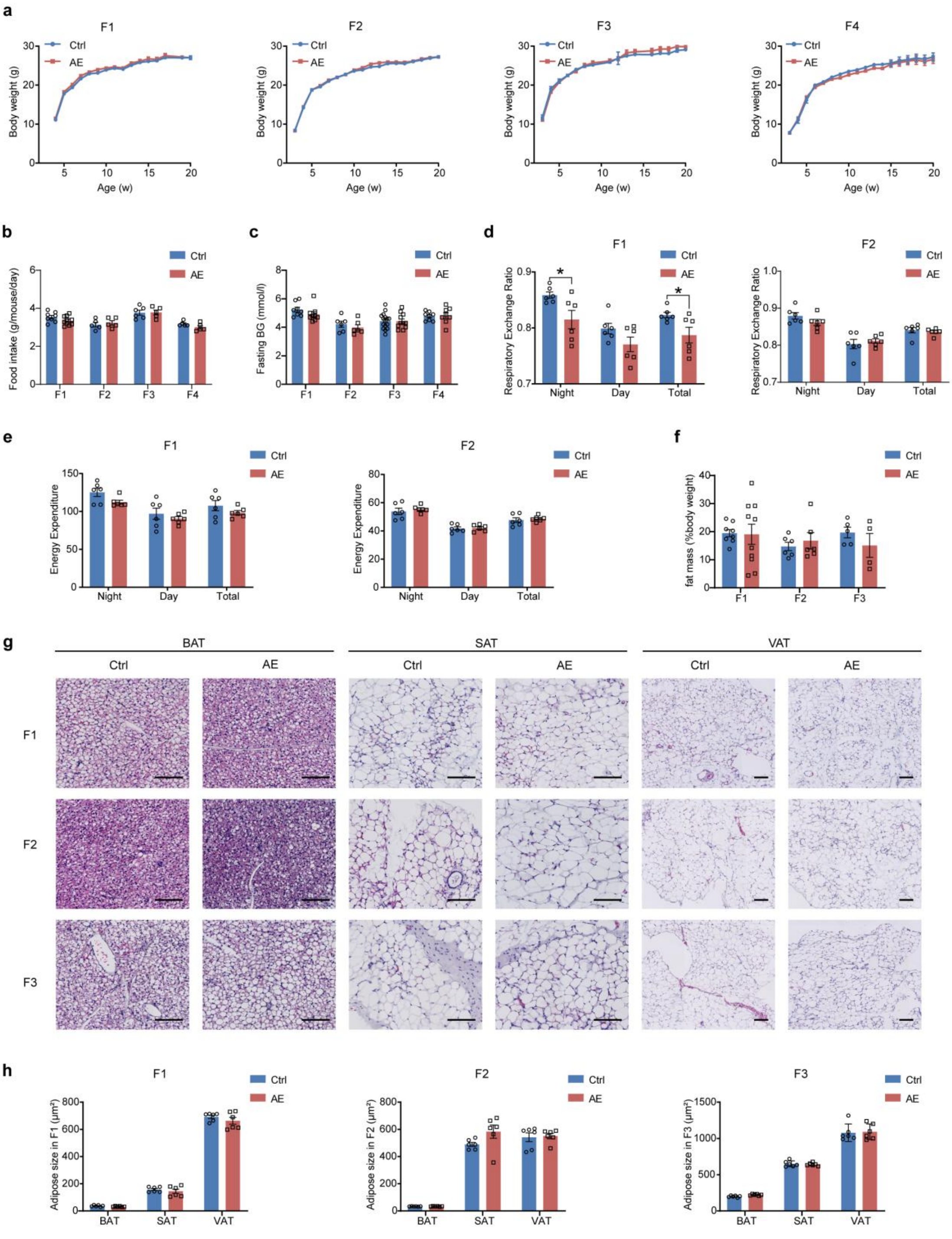

Supplementary Fig. S1 The effects of maternal androgen exposure on whole-body energy metabolism of male offspring.

a Body weight trajectories of F1–F4 male offspring without (Ctrl) or with maternal androgen exposure (AE). Ctrl (F1: *n* = 10; F2: *n* = 12; F3: *n* = 7; F4: *n* = 10); AE (F1: *n* = 12; F2: *n* = 13; F3: *n* = 8; F4: *n* = 12).

b The average food intake per day of F1–F4 male offspring. Ctrl (F1: *n* = 8; F2: *n* = 5; F3: *n* = 6; F4: *n* = 5); AE (F1: *n* = 10; F2: *n* = 5; F3: *n* = 5; F4: *n* = 5).

c Blood glucose levels upon 16 h of fasting in F1–F4 male offspring at 8 weeks of age. Ctrl (F1: *n* = 8; F2: *n* = 6; F3: *n* = 16; F4: *n* = 8); AE (F1: *n* = 9; F2: *n* = 6; F3: *n* = 11; F4: *n* = 8).

d, e Respiratory exchange ratio (**d**) and energy expenditure (**e**) were measured by indirect calorimetry in F1–F2 adult male offspring (*n* = 6).

f Body composition presented as percentage of fat mass normalized to body weight. Ctrl (F1: *n* = 8; F2: *n* = 6; F3: *n* = 5); AE (F1: *n* = 10; F2: *n* = 6; F3: *n* = 4).

g Representative images of adipocytes stained with hematoxylin and eosin (replicates of three mice per group). BAT: brown adipose tissue; SAT: subcutaneous adipose tissue; VAT: visceral adipose tissue. Scale bar: 100 μm.

h Adipocyte size of BAT, SAT, or VAT was quantified in F1–F3 male offspring.

Data are presented as mean ± SEM. Significance is assessed by two-tailed unpaired Student's *t*-test. *$P < 0.05$.

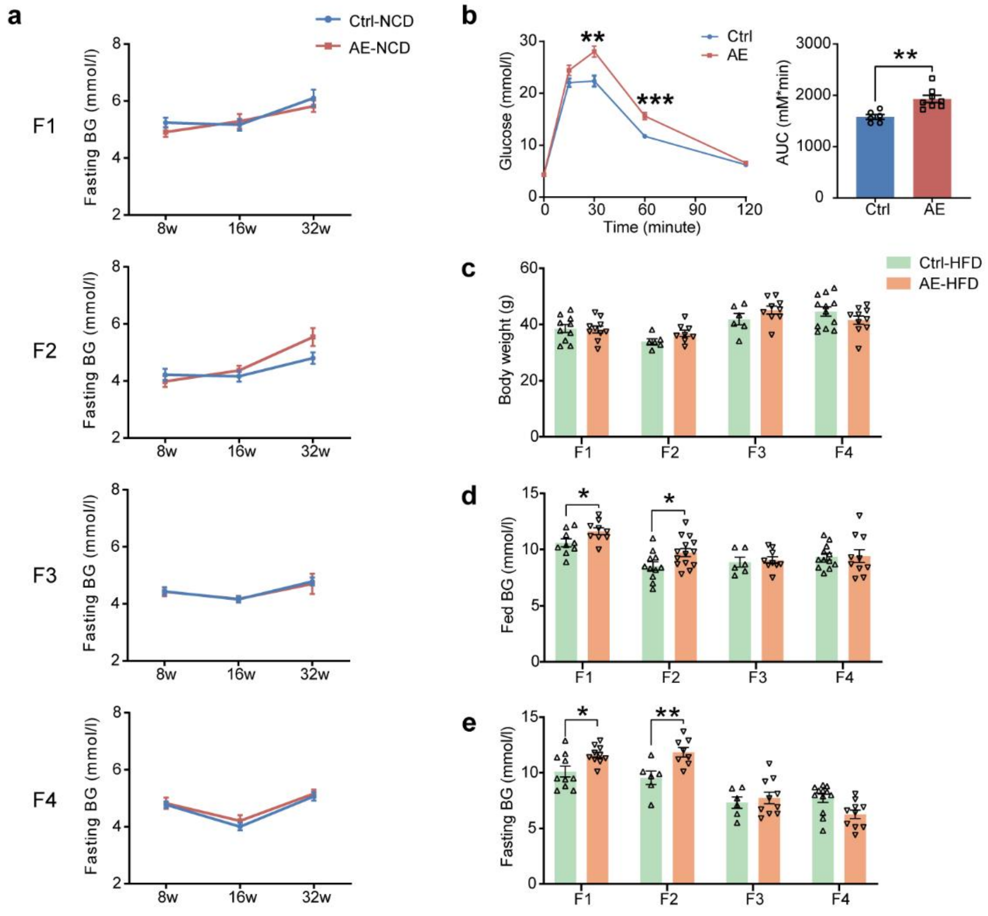

Supplementary Fig. S2 High-fat diet aggravates metabolic dysfunctions in male offspring with maternal androgen exposure.

a Blood glucose levels upon 16 h of fasting in F1–F4 male offspring at the indicated age under normal chow diet. Ctrl-8w (F1: *n* = 8; F2: *n* = 6; F3: *n* = 16; F4: *n* = 8); AE-8w (F1: *n* = 9; F2: *n* = 6; F3: *n* = 11; F4: *n* = 8); Ctrl-16w (F1: *n* = 9; F2: *n* = 8; F3: *n* = 7; F4: *n* = 10); AE-16w (F1: *n* = 11; F2: *n* = 9; F3: *n* = 7; F4: *n* = 14); Ctrl-32w (F1: *n* = 5; F2: *n* = 5; F3: *n* = 6; F4: *n* = 10); AE-32w (F1: *n* = 6; F2: *n* = 5; F3: *n* = 6; F4: *n* = 8).

b IPGTT of F2 female offspring and the corresponding area under the curve (AUC) of glucose levels (Ctrl: *n* = 6; AE: *n* = 8).

c Body weight of F1–F4 male offspring after high-fat diet (HFD) for 12 weeks. Ctrl-

HFD (F1: $n = 10$; F2: $n = 6$; F3: $n = 6$; F4: $n = 12$); AE-HFD (F1: $n = 10$; F2: $n = 8$; F3: $n = 9$; F4: $n = 10$).

d Fed blood glucose levels in F1–F4 male offspring under HFD. Ctrl-HFD (F1: $n = 9$; F2: $n = 11$; F3: $n = 6$; F4: $n = 12$); AE-HFD (F1: $n = 9$; F2: $n = 14$; F3: $n = 9$; F4: $n = 10$).

e Blood glucose levels upon 16 h of fasting in F1–F4 male offspring under HFD. Ctrl-HFD (F1: $n = 10$; F2: $n = 6$; F3: $n = 6$; F4: $n = 12$); AE-HFD (F1: $n = 10$; F2: $n = 8$; F3: $n = 10$; F4: $n = 10$).

Data are presented as mean ± SEM. Significance is assessed by two-tailed unpaired Student's *t*-test. $^{*}P < 0.05$, $^{**}P < 0.01$, $^{***}P < 0.001$.

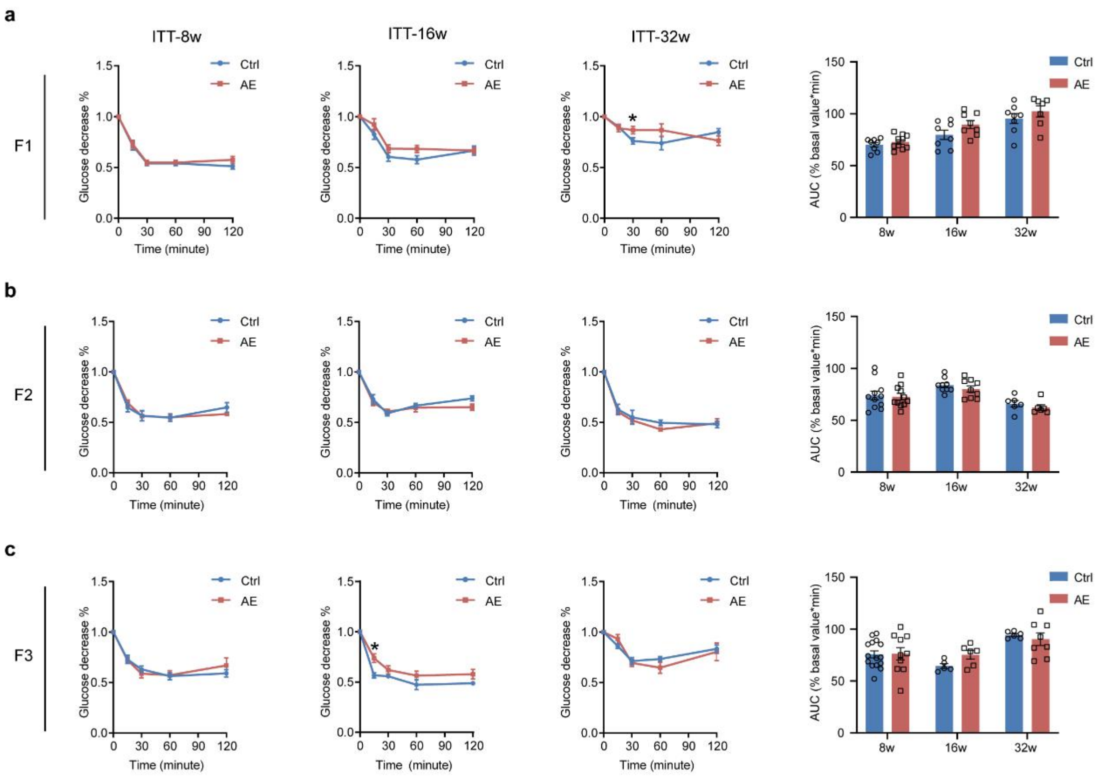

Supplementary Fig. S3 Maternal androgen exposure has limited impact on insulin sensitivity of F1–F3 male offspring.

a ITT and the corresponding AUC of F1 male offspring at the indicated age. 8 weeks (Ctrl: *n* = 8; AE: *n* = 10); 16 weeks (Ctrl: *n* = 8; AE: *n* = 8); 32 weeks (Ctrl: *n* = 8; AE: *n* = 7).

b ITT and the corresponding AUC of F2 male offspring at the indicated age. 8 weeks (Ctrl: *n* = 11; AE: *n* = 11); 16 weeks (Ctrl: *n* = 9; AE: *n* = 9); 32 weeks (Ctrl: *n* = 6; AE: *n* = 6).

c ITT and the corresponding AUC of F3 male offspring at the indicated age. 8 weeks (Ctrl: *n* = 15; AE: *n* = 10); 16 weeks (Ctrl: *n* = 5; AE: *n* = 6); 32 weeks (Ctrl: *n* = 6; AE: *n* = 8).

Data are presented as mean ± SEM. Significance is assessed by two-tailed unpaired

Student's *t*-test. *$P < 0.05$.

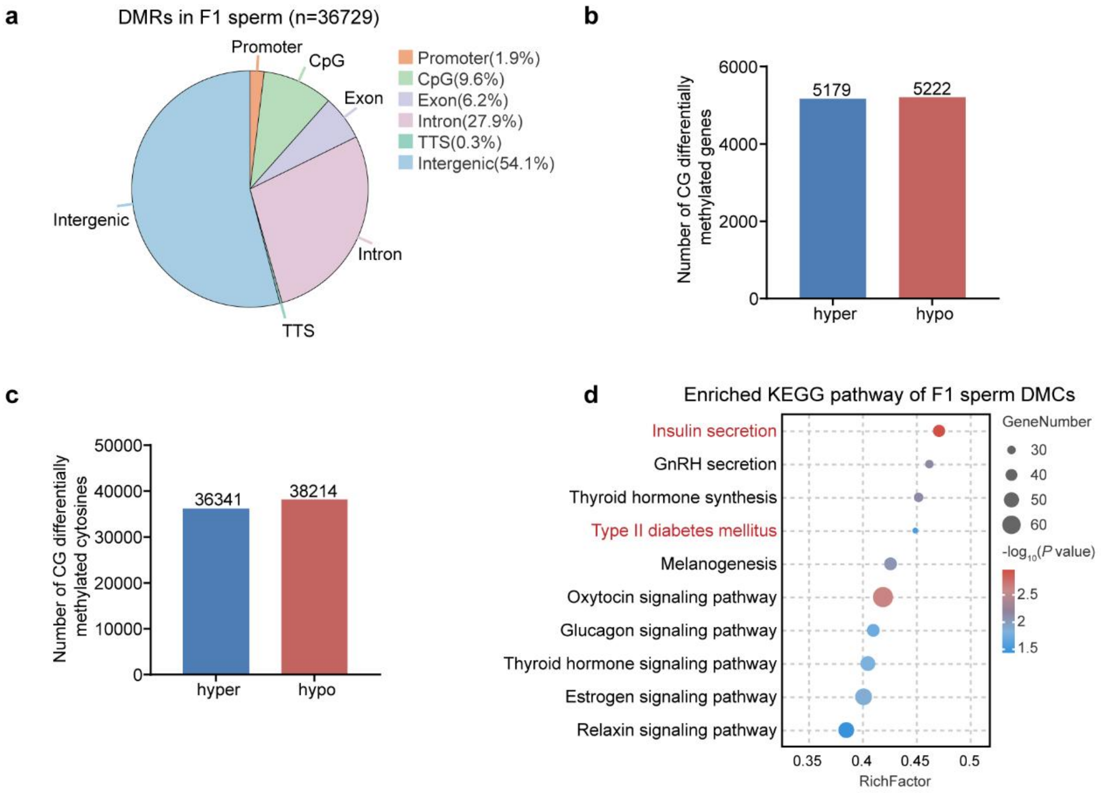

Supplementary Fig. S4 Analyses of the differentially methylated regions and cytosines in sperms of F1 mice.

a Distribution of the differentially methylated regions (DMRs) in F1 sperms across various genomic features. For regions belonging to more than one category, the classification followed the priority of promoter, exon, intron, and intergenic regions.

b The number of CG differentially methylated genes in F1 sperms.

c The number of CG differentially methylated cytosines in F1 sperms.

d Top 10 enriched metabolic pathways from KEGG analysis of genes with the differentially methylated cytosines (DMCs) in F1 sperms.

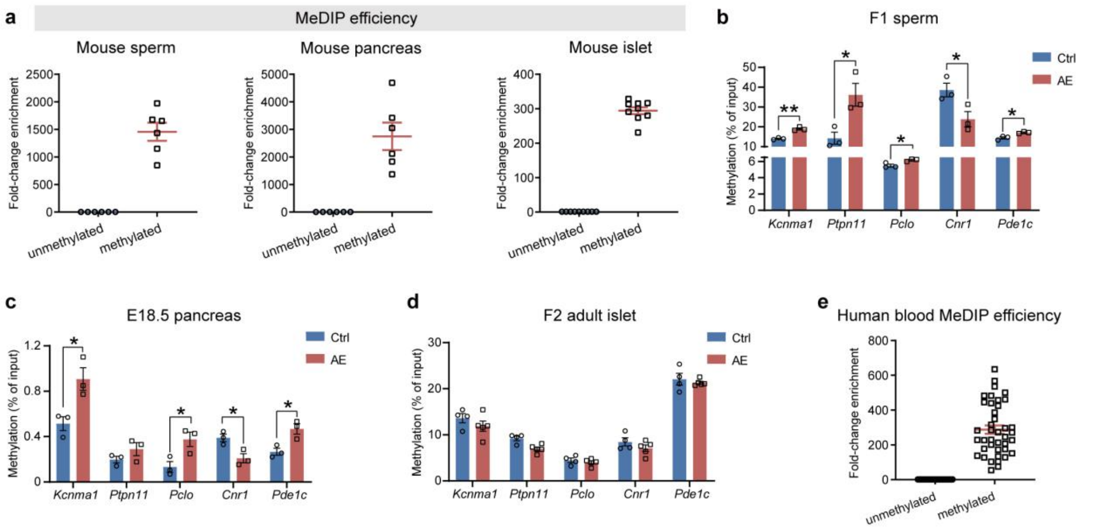

Supplementary Fig. S5 Transmission of aberrant DNA methylations from AE-F1 sperm to AE-F2 islets.

a MeDIP efficiency assessed in F1 sperms (n = 6), F2 E18.5 pancreases (n = 6), and F2 adult islets (n = 9) using spike-in controls of DNA methylated and unmethylated regions primers.

b MeDIP-qPCR analyses for DNA methylation levels of *Kcnma1*, *Ptpn11*, *Pclo*, *Cnr1*, and *Pde1c* in Ctrl-F1 and AE-F1 sperms (n = 3).

c MeDIP-qPCR analyses for DNA methylation levels of *Kcnma1*, *Ptpn11*, *Pclo*, *Cnr1*, and *Pde1c* in Ctrl-F2 and AE-F2 E18.5 pancreases (n = 3).

d MeDIP-qPCR analyses for DNA methylation levels of *Kcnma1*, *Ptpn11*, *Pclo*, *Cnr1*, and *Pde1c* in Ctrl-F2 (n = 4) and AE-F2 adult islets (n = 5).

e MeDIP efficiency assessed in human blood samples (n = 40) using spike-in controls of DNA methylated and unmethylated regions primers.

Data are presented as mean ± SEM. Significance is assessed by two-tailed unpaired Student's t -test. *P < 0.05, **P < 0.01.

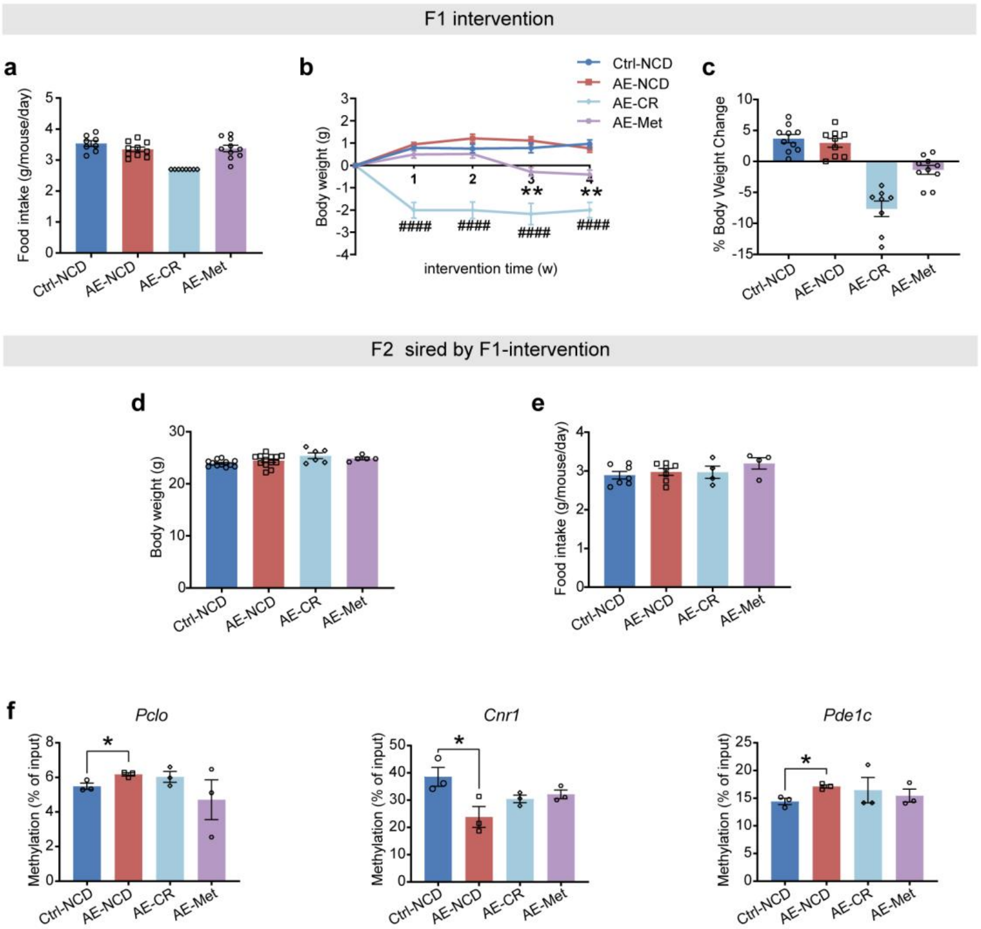

Supplementary Fig. S6 Effects of caloric restriction and metformin on F1 and F2 male offspring.

a The average food intake per day in F1 males from control group, AE group, AE group treated with caloric restriction (AE-CR), or AE group treated with metformin (AE-Met). An artificially restricted food intake in AE-CR group as 80% of ad libitum diet (Ctrl-NCD, *n* = 8; AE-NCD, *n* = 10; AE-CR, *n* = 8; AE-Met, *n* = 10).

b Body weight changes of F1 males after the interventions (Ctrl-NCD, *n* = 10; AE-NCD, *n* = 9; AE-CR, *n* = 8; AE-Met, *n* = 10).

c Percent of body weight change in AE-F1 males after the interventions (Ctrl-NCD, *n* = 10; AE-NCD, *n* = 9; AE-CR, *n* = 8; AE-Met, *n* = 10).

d Body weight of F2 male offspring derived from F1 males after the indicated interventions (Ctrl-NCD, $n = 12$; AE-NCD, $n = 13$; AE-CR, $n = 6$; AE-Met, $n = 5$).

e The average food intake per day in F2 male offspring derived from F1 males after the indicated interventions (Ctrl-NCD, $n = 7$; AE-NCD, $n = 7$; AE-CR, $n = 4$; AE-Met, $n = 4$).

f MeDIP-qPCR analyses for DNA methylation levels of *Pclo*, *Cnr1*, and *Pde1c* in sperms from Ctrl, AE, AE-CR, and AE-Met F1 male mice ($n = 3$).

Data are presented as mean ± SEM. *, Ctrl-NCD vs AE-NCD; statistical analyses are performed using two-tailed unpaired Student's *t*-test. #, AE-NCD vs AE-CR; statistical analyses are performed using one-way ANOVA with Tukey's multiple comparison test for other groups excluding Ctrl-NCD. *$P < 0.05$, **$P < 0.01$, ####$P < 0.0001$.
